# Microbial river-to-sea continuum: gradients in benthic and planktonic diversity, osmoregulation and nutrient cycling

**DOI:** 10.1186/s40168-021-01145-3

**Published:** 2021-09-20

**Authors:** Hwee Sze Tee, David Waite, Gavin Lear, Kim Marie Handley

**Affiliations:** 1 School of Biological Sciences, University of Auckland, Auckland, 1010 New Zealand; 2 Current address: Ministry for Primary Industries, Auckland, New Zealand

## Abstract

**Background:**

Coastal aquatic ecosystems include chemically distinct, but highly interconnected environments. Across a freshwater-to-marine transect, aquatic communities are exposed to large variations in salinity and nutrient availability as tidal cycles create periodic fluctuations in local conditions. These factors are predicted to strongly influence the resident microbial community structure and functioning, and alter the structure of aquatic food webs and biogeochemical cycles. Nevertheless, little is known about the spatial distribution of metabolic properties across salinity gradients, and no study has simultaneously surveyed the sediment and water environments. Here, we determined patterns and drivers of benthic and planktonic prokaryotic and microeukaryotic community assembly across a river and tidal lagoon system by collecting sediments and planktonic biomass at nine shallow subtidal sites in the summer. Genomic and transcriptomic analyses, alongside a suite of complementary geochemical data, were used to determine patterns in the distribution of taxa, mechanisms of salt tolerance, and nutrient cycling.

**Results:**

Taxonomic and metabolic profiles related to salt tolerance and nutrient cycling of the aquatic microbiome were found to decrease in similarity with increasing salinity, and distinct trends in diversity were observed between the water column and sediment. Non-saline and saline communities adopted divergent strategies for osmoregulation, with an increase in osmoregulation-related transcript expression as salinity increased in the water column due to lineage-specific adaptations to salt tolerance. Results indicated a transition from phosphate limitation in freshwater habitats to nutrient-rich conditions in the brackish zone, where distinct carbon, nitrogen and sulfur cycling processes dominated. Phosphorus acquisition-related activity was highest in the freshwater zone, along with dissimilatory nitrate reduction to ammonium in freshwater sediment. Activity associated with denitrification, sulfur metabolism and photosynthesis were instead highest in the brackish zone, where photosynthesis was dominated by distinct microeukaryotes in water (*Cryptophyta*) and sediment (diatoms). Despite microeukaryotes and archaea being rare relative to bacteria, results indicate that they contributed more to photosynthesis and ammonia oxidation, respectively.

**Conclusions:**

Our study demonstrates clear freshwater–saline and sediment–water ecosystem boundaries in an interconnected coastal aquatic system and provides a framework for understanding the relative importance of salinity, planktonic-versus-benthic habitats and nutrient availability in shaping aquatic microbial metabolic processes, particularly in tidal lagoon systems.

**Video abstract**

**Supplementary Information:**

The online version contains supplementary material available at 10.1186/s40168-021-01145-3.

## Introduction

Across connected riverine, estuarine and marine environments, freshwater flow and tidal influx helps to redistribute nutrients and establish a strong salinity gradient [1–3]. Such gradients directly influence the resident community composition and distribution, resulting in lower benthic macrofaunal species richness within the horohalinicum (where salinities are between 5 and 8 [4]) and higher microbial richness [5–8]. Global surveys of microbial diversity have demonstrated that both salinity and environment type (such as water or sediment) are major factors influencing bacterial [9] and archaeal community composition [10]. Besides nutrients, river discharge and phytoplankton community composition are also known to drive changes in planktonic estuarine communities [11, 12]. Previous studies of planktonic prokaryotic communities across freshwater-to-marine transects have highlighted significant differences in gene abundances related to glycolysis, respiration, catabolic pathways, osmolyte and metal transport, and the biosynthesis of quinones and isoprenoids between low and high salinities, in line with the shifts in taxonomic groups [13, 14]. However, little is known about the collective microbial ecology of bacteria, archaea and microeukaryotes in sediment and water across river-to-sea salinity gradients; how these factors (water, sediment, salinity) affect microbial gene expression across this environmental continuum also remains largely unexplored.

A key factor shaping microbial community distributions across salinity gradients is lineage-specific adaptations for salt tolerance [6, 8–10, 12, 15, 16]. Bacteria adopt two distinct strategies to cope with osmolarity changes in their environment: (1) a salt-in strategy which promotes K^+^ accumulation and (2) the synthesis or uptake of organic compatible solutes which function as osmoprotectants [17]. Various K^+^ uptake systems have been reported to date, for example, both *trkA* and *kup* systems in hyperosmotic environments, and the *kdp* system for low external K^+^ concentrations [18–21]. Bacteria can also employ various Na^+^/H^+^ antiporters and Na^+^ translocating dehydrogenases to export Na^+^ extracellularly in highly saline environments [13, 15]. Taxa and strategies employed are therefore predicted to change across coastal salinity gradients. Although, certain lineages, for instance, members of the order *Pelagibacterales* (SAR11), have been found at different salinities, suggesting ecological adaptations within microbial lineages to salt [16, 22].

In addition to salinity, dramatic differences in nutrient availability and types are typically observed across freshwater to marine systems and have been predicted to strongly influence community composition and function [1–3]. These systems mix nitrogen-rich freshwater with sulfate and phosphate-rich marine water [23, 24]. Sulfate-rich saline water promotes the formation of ferrosulfides, resulting in less phosphate sorption in sediments and higher phosphate concentrations in saline water [23, 25, 26]. Riverine systems, however, are often phosphate-limited due to greater phosphate sorption in non-saline sediments [27]; it is therefore likely that freshwater microbial communities rely on high-affinity phosphate uptake mechanisms. Estuarine conditions are comparatively rich in both nitrogen and phosphorus, which support the growth of primary producers [28], and in turn, heterotrophs [29, 30]. Benthic photosynthesis has been shown to promote nutrient-regulating biogeochemical processes in estuaries, such as coupled nitrification–denitrification [31]. However, the relative contribution of each of the taxa within the microbiome toward biogeochemical cycling across river–estuary–marine transects remains understudied.

To determine microbial taxonomic and functional diversity, and metabolic hotspots, across a river-to-sea transect, we sampled benthic sediment (hereafter referred to as “benthic” samples) and overlying water (“planktonic” samples) along a 5-km stretch connecting the Waiwera river, estuary and beach (Auckland, New Zealand). The Waiwera river predominantly drains rural pastoral catchments [32], and is therefore impacted by anthropogenic activities, as is typical for estuaries globally [33, 34]. The river and estuary have a water depth of approximately 0.2–1 m, and the estuary is classified as a permanently open tidal lagoon estuary [35] and is subjected to daily tidal influxes from the Hauraki Gulf (Pacific Ocean). Tidal lagoon systems, also known as bar-built coastal lagoons, or barrier enclosed lagoons, can be found throughout the world (e.g. Laguna Madre in Texas, USA [36]; Mosquito Lagoon in Florida, USA [37]; and Paravur estuary in Kerala, India [38]) and occupy about 13% of the global coastline [39]. They are also the most common estuary systems in New Zealand [40] and the UK [41]. A natural continuous salinity gradient occurs along the Waiwera river and estuary, potentially exerting a strong influence on the assembly of microbial communities. We hypothesised that non-saline, saline, water and sediment communities would be strongly demarcated by distinct prokaryotic and eukaryotic compositions, and utilise distinct osmoregulatory and nutrient acquisition mechanisms, reflecting, for example, nutrient limitations in the freshwater (P) and marine (N) end-member environments. Combining metagenomic, metatranscriptomic and geochemical data, we identified the impact of salinity and nutrient availability on prokaryotic and microeukaryotic community diversity and on critical physiological and ecological processes, such as osmoregulation, primary production and nutrient cycling.

## Results and discussion

### Community partitioning based on salinity and sediment–water habitat preferences

#### Contribution of salinity versus other environmental parameters in shaping microbial communities

**Fig. 1:**
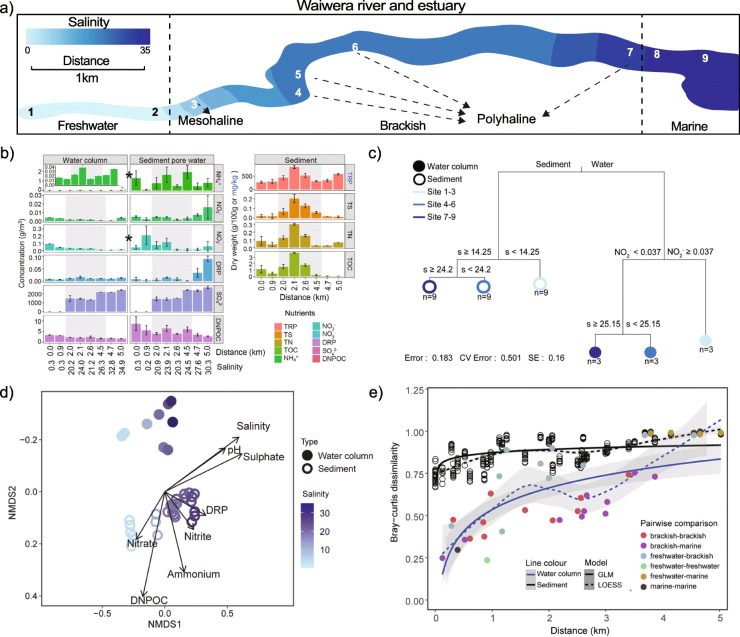
Plots showing variation in nutrient concentrations and community composition. **a** The Waiwera river sampling locations (1 to 9) with water column salinity gradient (0–35) shown using kriging interpolation. **b** Nutrient concentrations in sediment, sediment pore water and water samples taken in triplicate across the sampling sites. Grey background represents brackish sites (2.0–4.5 km along the transect). Abbreviations: TRP total recoverable phosphorus, TS total sulfur, TN total nitrogen, TOC total organic carbon, DRP dissolved reactive phosphorus, DNPOC dissolved non-purgeable organic carbon. Error bars represent standard errors of means. Asterisks (∗) indicate a significant difference between all water column and sediment samples (Wilcoxon *p* < 0.05). **c** Multivariate regression tree of microbial community abundance data associated with the environmental variables. Abbreviations: s salinity, NO_2_^−^ nitrite, *n* sample size. Units: nitrite (g/m3). **d** NMDS ordination of small subunit rRNA gene sequences based on Bray–Curtis dissimilarities. Samples are coloured according to salinity. Environmental vectors were fitted onto the NMDS scores of the microbial community by the R-function envfit (*p* < 0.05, permutation = 999). **e** Distance–decay relationships of community dissimilarity (Bray–Curtis index) with increasing geographical distance. The regression lines were fitted to the data points using log generalised linear (GLM, solid line) and LOESS (dotted line) models. Each data point represents a pairwise comparison of samples from the water column (closed circles) or sediment (open circles)

Nutrient and salinity gradients are major features of estuarine systems, leading to higher estuarine productivity [1–3]. Accordingly, we observed a general decrease in nitrate concentrations along the Waiwera river–estuary–sea transect, higher organic carbon concentrations in brackish sediments, and an increase in dissolved reactive phosphorus and sulfate with salinity (Fig. 1a, b). We therefore tested the contribution of salinity relative to other geochemical variables in differentiating prokaryotic and microeukaryotic composition along this transect. To achieve this, near full-length small subunit (SSU) rRNA gene sequences were assembled and clustered into 6964 bacterial, 291 microeukaryal and 79 archaeal operational taxonomic units (OTUs, unique at 97% sequence identity). Results showed salinity best explains differences in sediment bacterial and microeukaryal community composition (Spearman’s correlation coefficient *ρ* = 0.43–0.91) and planktonic bacterial and microeukaryal composition (*ρ* = 0.78–0.91, Supplementary Table 1). Two subsets of parameters were maximally correlated with dissimilarities among planktonic (salinity, ammonium, and phosphate, ρ = 0.19) and benthic archaea (salinity and nitrate, *ρ* = 0.90, Supplementary Table 1), suggesting that besides salinity, nutrients play important roles in structuring these aquatic communities, in agreement with previous studies [11, 12, 22]. Overall, our results correlate strongly with research showing salinity is a major global driver of prokaryotic community composition [9, 10].

Although salinity strongly differentiated each of the planktonic and benthic communities relative to other geochemical factors, water-vs-sediment environment type was found to be the single most important factor distinguishing microbial communities overall (Fig. 1c). This was due to consistently large differences in benthic and planktonic communities across the gradient, regardless of salinity (Fig. 1d; Supplementary Table 2), which resulted in significantly different benthic and planktonic microbial communities overall based on Bray–Curtis dissimilarities (Benjamini–Hochberg adjusted *p* < 0.05, Supplementary Table 3; Fig. 1d and Supplementary Figure 1). Results, therefore, support research highlighting the importance of these environment types, along with salinity, in structuring microbial communities [9, 10]. However, few studies, as here, have compared sediment and water communities directly within the same system [42, 43]. We found that only when comparing the effect of end-member salinities (non-saline vs marine) on benthic and planktonic communities were differences in microbial community composition comparable to those between water and sediment at the same salinity (Bray–Curtis dissimilarities 0.97 to 0.99 on average; Supplementary Table 2). Predictably, the combination of salinity and environment type contributed to almost entirely distinct communities (Bray–Curtis dissimilarities of 0.99 to 1.0, or 1.0 on average). Sediment and water environments are typically separated by large differences in redox processes, oxygen, nutrient and terminal electron acceptor availability [44, 45]. This is also evident in our study, where concentrations of ammonium, nitrate and dissolved non-purgeable organic carbon (DNPOC) were significantly higher in the sediment porewater, compared with the overlying water column (Wilcoxon, *p* < 0.05, Fig. 1b).

#### Salinity gradients and the non-saline–saline divide in sediment and water microbial communities

**Fig. 2:**
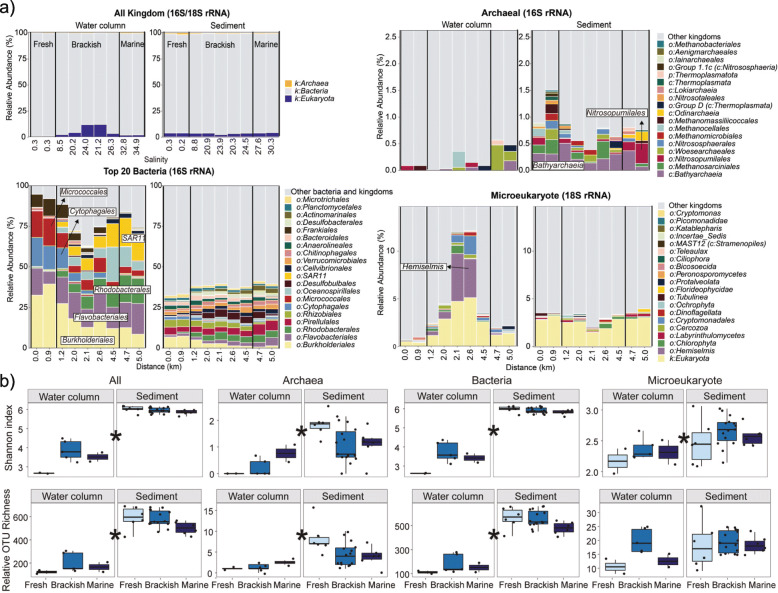
Community distribution and alpha diversity. **a** Bar plots indicate relative abundances of EMIRGE-reconstructed 16S and 18S rRNA gene sequences. Site salinities are shown in the upper left plots, and transect distances are given in the lower plots. Taxa are coloured at the order level. For each plot, samples from the water column (left) and sediment (right) are further categorised into freshwater, brackish or marine, as indicated in the top left plot. **b** Boxplots illustrate Shannon indices and relative OTU richness in both sediment and water column environments across the salinity gradient for archaeal, bacterial and microeukaryal communities (*n* = 36). Colours indicate environment type (freshwater, brackish and marine) based on the salinity gradient. Boxes represent the interquartile range (IQR) between the first and third quartiles and the horizontal line inside the box represents the median. Whiskers represent the lowest and highest values within 1.5 times the IQR from the first and third quartiles, respectively. Individual sample values are shown as dots. Asterisks (^∗^) indicate a significant difference between all water column and sediment samples (Wilcoxon *p* < 0.05)

Consistent with salt as a major driver of aquatic community composition, reconstructed SSU rRNA and Braken2-estimated species abundance profiles indicated large differences in the composition of bacteria, archaea and microeukaryotes across the salinity gradient within sediment and water environments, although bacteria invariably dominated both the benthic and planktonic communities (Fig. 2a, trends exhibited by key taxa are described in Supplementary Information). An overall decrease in both planktonic and benthic community similarities occurred with increased difference in salinity and geographic distance (Fig. 1e and Supplementary Figure 2a), resulting in a clear separation between non-saline and saline community compositions (Fig. 1d). Results reflect the substantial physiological adaptations required for salt tolerance [14–16]. The rate of increasing dissimilarity was far greater among water column communities (analysis of covariance, *p* < 0.0001), owing to the relatively high similarity between neighbouring communities. Greater spatial dissimilarity overall was observed among communities at benthic sites, including among sites located only 5 m apart (Fig. 1e). This suggests sediment is a more heterogeneous environment compared to the overlying water, and is consistent with previous comparisons of planktonic and benthic microbial communities in river or coastal settings [42, 43]. The strong spatial heterogeneity of benthic communities is likely due to greater stability within sediments, which are less impacted by tidal mixing [1, 46].

To further compare community differences across the transect, we categorised sediment and overlying water samples as each belonging to three distinct environments (non-saline, 0–0.5; brackish, 0.5–30; marine, 30–35), based on the salinity of porewater or water samples, respectively (Fig. 1a and Supplementary Table 4) [47]. The benthic community composition (based on OTU abundances) was significantly different among non-saline, brackish and marine environments (Benjamini–Hochberg adjusted *p* < 0.05, Supplementary Table 3). Fewer benthic and planktonic OTUs were shared between freshwater and brackish (6.4–10.9%) than between saline brackish and marine (13.5–17.9%) environments (Supplementary Figure 2b). This is reflected by the planktonic distance–decay curve, which shows greater dissimilarity at the freshwater–brackish transition (peak at ~1.5 km) and higher similarity at the brackish–marine transition (trough at ~2.5 km; Fig. 1e). These results support findings that salinity strongly partitions freshwater and marine planktonic prokaryotic communities [14, 22], and also demonstrate that the relatively small increase in salinity (of 8) spanning the freshwater–saltwater transition represents a powerful ecological barrier for both benthic and planktonic communities.

#### Influence of salinity on microbial alpha diversity

Microbial community alpha diversity, measured via the Shannon index and relative OTU richness, was significantly higher in sediments across the entire transect (Fig. 2a; Wilcoxon, *p* < 0.001), likely due to greater nutrient availability (Fig. 1b), availability of terminal electron acceptors, greater diversity in environmental niche space and greater community stability associated with more static environmental conditions [1, 46, 48]. Benthic and planktonic communities exhibited opposing trends in richness and Shannon diversity across the salinity gradient (Fig. 2b). In contrast to previous planktonic studies that showed no clear trend in diversity [6, 7] or a reduction in diversity with increasing salinity [11, 12], the median taxon richness increased from a minimum in freshwater to a maximum in marine water (123 to 166 OTUs), while the Shannon index increased from 2.7 in freshwater to 3.5–3.8 in brackish and marine water. Sediment community Shannon diversity and richness decreased along this gradient (Shannon indices from 6.0 to 5.9; OTUs from 596 to 502). While greater diversity or taxon richness in marine water versus freshwater may be attributed to OTUs being flushed out to sea, we observed no evidence for this, as very few OTUs (<3%) were shared between these systems (Supplementary Figures 2b and 3). The same patterns were observed when considering specific lineages (archaea, bacteria and microeukaryotes), except benthic microeukaryotes displayed no clear trend (Fig. 2b). Results showed no obvious reduction in prokaryotic or microeukaryote richness in the brackish zone, in contrast with Remane’s species minimum concept, which proposes minimum richness for benthic macrofauna in the brackish horohalinicum [4]. Our results on prokaryotes and microeukaryotes, taken together with other studies that have found a protistan species-maximum in brackish water [5, 49], suggest essential differences in taxa diversity associated with salinity gradients.

### Osmoregulation, nutrient metabolism and primary production across environments

#### Differences in functional gene abundance and expression across sampled gradients and relationship to taxa composition

**Fig. 3:**
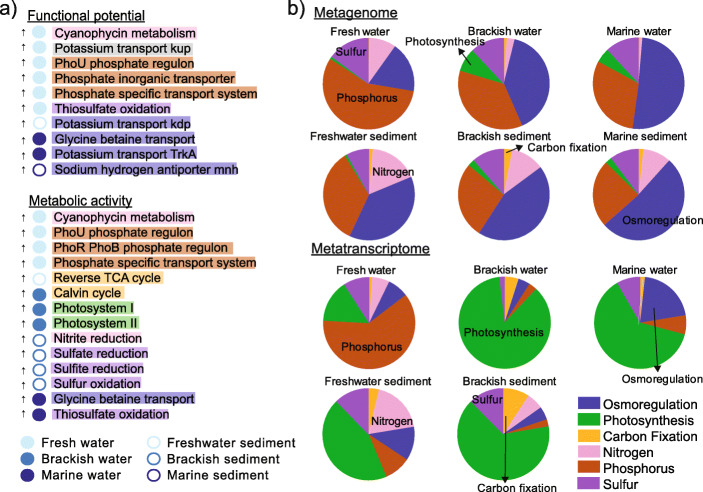
Discriminative features and distribution of functional potential and metabolic activity across the salinity gradient. **a** Differentially abundant metabolic functions from metagenomic (top, *n* = 36) and metatranscriptomic (bottom, *n* = 33) data, as determined by LEfSe (specific pathways from Supplementary Table 5). Arrows indicate functions were significantly over-represented in their specific habitat. **b** Pie charts showing the relative fraction of functional gene categories across the freshwater-to-marine gradient based on metagenome and metatranscriptome data. Transcriptomic data were not obtained for marine sands due to low RNA quality. Colours in both panels indicate key nutrient acquisition and osmoregulation pathways

Overall, metabolic profiles (genes and genes expressed) decreased in similarity with increasing salinity divergence (Supplementary Figure 2c). However, neither taxonomic nor functional gene data were strong indicators of gene expression, as previously observed for freshwater-to-marine planktonic [14], ocean [50] and hypersaline desert environments [51]. Mantel tests showed that while taxonomic beta-diversity was strongly correlated with differences in functional potential (*ρ* = 0.90, *p* < 0.01), it was only weakly correlated with differences in gene expression (*ρ* = 0.54, *p* < 0.01). Similarly, we identified a moderate correlation between differences in gene and transcript abundances (*ρ* = 0.70, *p* < 0.01). Microbial composition is thought to be a poor predictor of ecological processes due to functional redundancy and variations in environmental response, leading to marked differences in transcript expression [50–53]. To ascertain which metabolic features, associated with osmoregulation, nutrient metabolism and primary production, were strongly differentiated among freshwater, brackish and marine environments, and between water and sediment, we applied linear discriminant analysis effect size (LEfSe) and weighted gene co-expression network analysis (WGCNA). LEfSe results revealed 25 discriminative features from the metagenomes and 42 from the metatranscriptomes (Fig. 3a; Kruskal–Wallis *p* < 0.05, Supplementary Table 5), indicating a large shift in metabolic potential and, most notably, activity across this transect (Supplementary Figure 4). In agreement with LEfSe results, WGCNA analysis showed 4 out of 6 co-expressed gene clusters were significantly associated with environment (Supplementary Figure 5; details are described in Supplementary Information), revealing a clear division in metabolism related to nutrient acquisition and osmoregulation between sediment and water and between saline and non-saline environments. Genes and transcript abundances related to phosphorus metabolism, photosynthesis and osmoregulation were significantly higher in fresh, brackish and marine water, respectively (Fig. 3 and Supplementary Figure 5). In contrast, brackish sediment was characterised by significantly higher sulfur metabolism (Fig. 3a and Supplementary Figure 5). Nutrients in tidal lagoon or bar-built estuaries, such as in this study, are generally derived from runoff from land or groundwater inputs, and promote primary productivity and nutrient regeneration in brackish systems [54]. Fewer genes and transcripts were identified as significantly and differentially abundant among benthic environments due to large variations in their relative abundance, reflecting the high spatial heterogeneity in benthic community composition and nutrient availability (Figs. 1b and 2a).

#### Distinct non-saline and saline osmoregulation strategies

**Fig. 4:**
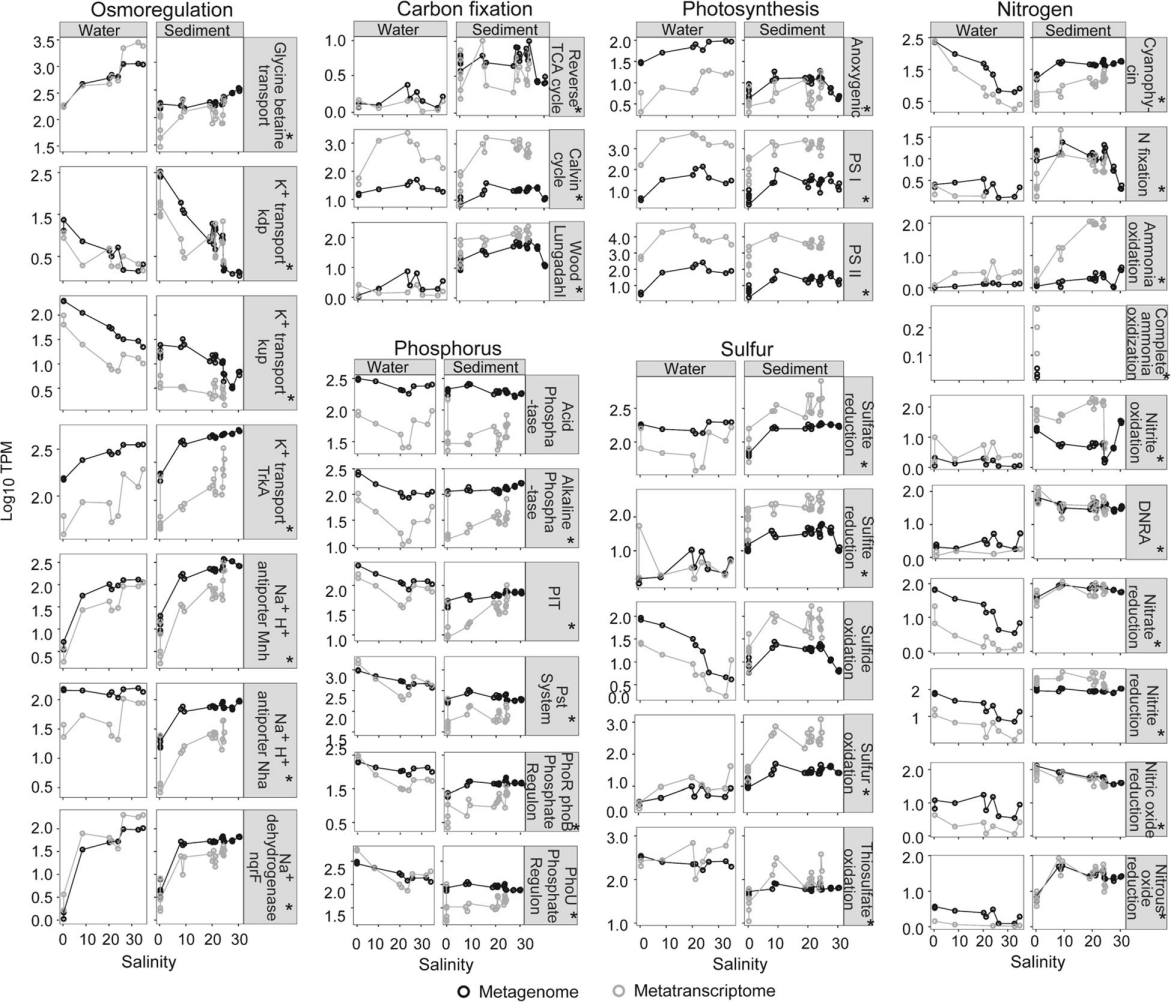
Gene and gene expression profiles across the salinity gradient. Line plots showing log_10_-normalised gene and transcript abundances related to osmoregulation, nutrient acquisition and primary production pathways across the salinity gradient. Genes per category are given in Supplementary Table 9. Asterisks (*) indicate significant correlations between metagenomes and metatranscriptomes (Spearman *ρ* > 0.5, *p* < 0.05). Abbreviations: TPM genes/transcripts per million mapped reads

**Fig. 5:**
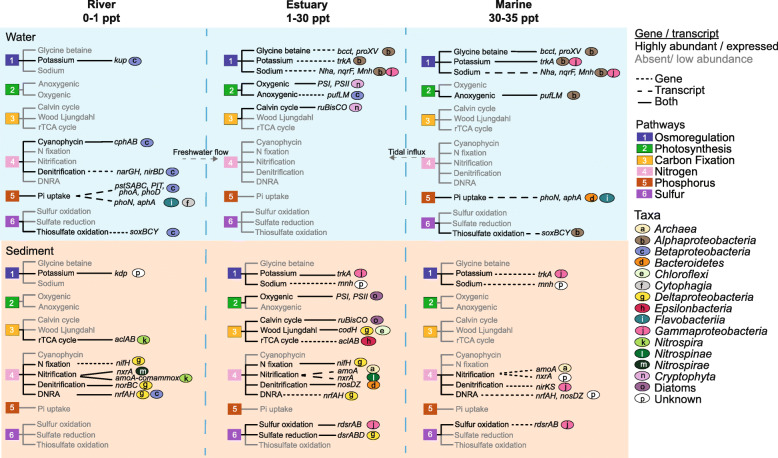
Schematic illustrating metabolic genes and gene expression in benthic and planktonic environments across the freshwater-to-marine transect. Genes with relative abundances and expression levels at least twice higher than in those of other environments are shown. No transcriptomics data were available for marine sediments at sites 8 and 9. Key on right: rectangles with numbers represent metabolic pathways; ovals with letters indicate the main taxa expressing these genes

In line with previous research indicating that microbial taxonomic distributions are primarily driven by salinity [14], largely due to lineage-specific adaptations to salt tolerance [15, 17, 55], our results show that dominant microbial taxa in non-saline (*Betaproteobacteria*) and saline (*Alphaproteobacteria* and *Gammaproteobacteria*) habitats adopted distinct osmoregulation strategies (Figs. 4 and 5 and Supplementary Figure 6). While K^+^ acquisition and Na^+^ export are considered important mechanisms for osmoregulation in brackish or marine environments [14, 15], results here illustrate that K^+^ transport is also crucial in freshwater communities. We observed a significantly greater abundance of the potassium (K^+^) transporter gene *kdp*, in non-saline sediment (Kruskal–Wallis, *p* < 0.05, Fig. 4 and Supplementary Figure 4). In contrast, *trkA* was more abundant in marine water and sediment (Fig. 4). The *trkA* system is prominent under hyperosmotic stress [19], characteristic of the marine environment. The repressible high-affinity K^+^ transporter, *kdp*, is known to be active at low external K^+^ concentrations [21], consistent with freshwater environments.

We found a significantly higher abundance of Na^+^/H^+^ antiporter genes (*mnhABCDEFG*) in marine sediment, and higher expression of Na^+^ translocating dehydrogenase (*nqrF*) and glycine betaine transporter (*bcct* and *proXV*) genes in marine water (Figs. 3a and 4, Supplementary Figure 4), primarily contributed by *Alphaproteobacteria* and *Gammaproteobacteria*, with an apparent increase in gene expression starting from site 7 (salinity = 26; Supplementary Figure 6). Glycine betaine transporter and Na^+^/H^+^ antiporter gene expression in the water was also significantly correlated with salinity (*ρ* = 0.90–0.95, *p* < 0.01, Supplementary Figure 6). In the marine environment, some bacteria rely on Na^+^-motive force to routinely export Na^+^ via various Na^+^/H^+^ antiporters and Na^+^ translocating dehydrogenases [56–59]. Increased abundance and expression of Na^+^ transporter genes with salinity suggests that brackish and marine microorganisms increasingly exported destabilising Na^+^ in exchange for greater accumulation of K^+^ within cells, which stabilise acidic salt-adapted proteins [17, 60, 61]. Glycine betaine transporters, in contrast, uptake organic solutes to achieve osmotic equilibrium [17, 62], or for metabolism and ATP generation [63].

#### Spatial distribution of light and dark primary production

**Fig. 6:**
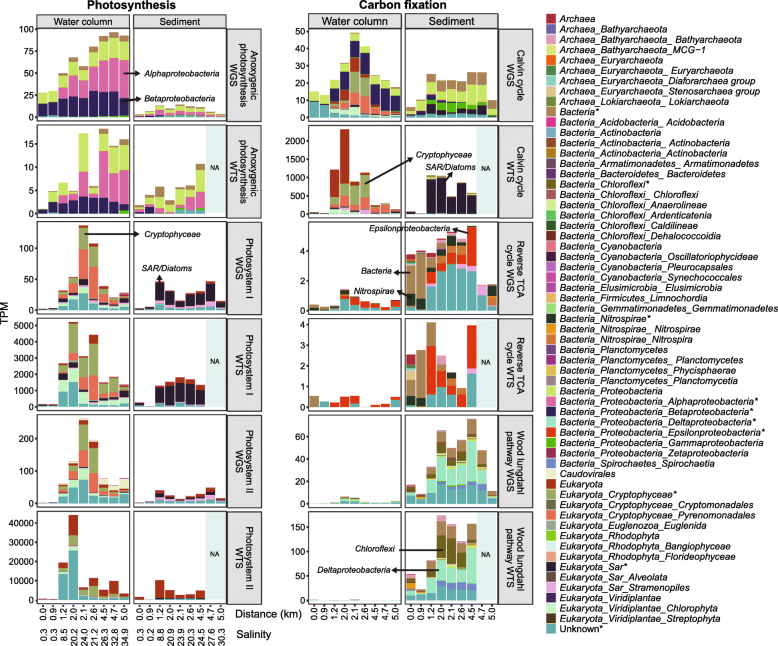
Distribution of taxon-specific genes and transcripts involved in photosynthesis and carbon fixation across the study sites. NA indicates that no transcriptomic data are available for marine sediments. Abbreviations: WGS metagenome, metatranscriptome (WTS), genes/transcripts per million mapped reads (TPM)

Autotrophic microbial and microeukaryotic communities play crucial roles in aquatic primary production and form the base of the aquatic food chain. We observed taxonomically diverse organisms across the river–marine transect with capabilities for oxygenic or anoxygenic photosynthesis and carbon fixation via the Calvin–Benson, Wood–Ljungdahl and reverse tricarboxylic acid (TCA) pathways (Figs. 5 and 6). Gene expression related to primary production was the highest in water in the mid-brackish zone (salinity = 24; Figs. 4 and 6). This corresponded with increased phytoplankton relative abundance, particularly *Cryptophyta* (genus *Hemiselmis*; Figs. 2a and 6), and a peak in ammonium (0.04 g/m^3^, Fig. 1b)—the preferred nitrogen source for *Cryptomonads* [64] (Supplementary Information). Data indicate phytoplankton actively assimilated carbon via the Calvin–Benson cycle (RuBisCO) and conserved energy via oxygenic photosynthesis (photosystems I and II; Figs. 5 and 6). In contrast, diatoms can photosynthesise and fix carbon efficiently under low light [65]. Coastal lagoon systems, including the Waiwera river, are shallow water bodies (<5-m depth) [66], where light can penetrate to the bottom sediments and promote benthic primary productivity [54]. Consistent with this, Waiwera had a low median river turbidity of 7.0 Nephelometric Turbidity Units (NTU) reported for the sampling year (2018, https://www.lawa.org.nz/), and results show that diatoms dominated sediment primary production across the brackish transect (Fig. 6). Increased phytoplankton primary production, and consequently, sinking biomass, may explain the higher concentrations of total sedimentary organic carbon, nitrogen and phosphorus measured at the mid-brackish site (Fig. 1b). High benthic primary production (Figs. 3b and 6) and mud content may also contribute to higher organic carbon in brackish sediment [67–70] (average 17.8% versus 4.3% elsewhere, Supplementary Table 4).

There is limited information on carbon fixation pathways utilised across salinity gradients, but a few studies show that estuaries are global hotspots for carbon cycling and assimilation [28, 71, 72]. Here, the Calvin cycle, in conjunction with photosynthesis, was significantly more highly expressed in brackish water (significant, LDA score ≥ 2), whereas genes related to dark carbon fixation, such as the reductive TCA cycle and Wood–Ljungdahl pathway, were primarily expressed in sediment environments. While we did not measure dissolved oxygen in this study, microorganisms undertaking dark carbon fixation typically thrive across a redox gradient, such as the oxic–anoxic interface in aquatic sediments [73, 74]. Both the Wood–Ljungdahl pathway and reductive TCA cycle are strongly associated with anaerobes, although the latter is also found in microaerobes and some aerobes [75, 76]. Among organisms undertaking dark carbon fixation, we found that the reverse TCA cycle genes (*aclA*) were significantly more highly expressed in non-saline sediment (LDA score ≥2), and predominantly by *Nitrospira* (Fig. 6), which our results indicate also undertook complete ammonia oxidation (comammox) in this environment (Fig. 5). In contrast, Wood–Ljungdahl pathway *codh* genes were highly expressed in brackish sediments, primarily by *Chloroflexi* and *Deltaproteobacteria* (Figs. 5 and 6). Wood–Ljungdahl pathway gene expression in the brackish sites was at least 100 times greater than reverse TCA cycle gene expression, but 10 times less than the Calvin cycle gene expression (Figs. 5 and 6), highlighting the significance of the Calvin cycle and Wood–Ljungdahl pathway for carbon assimilation in this brackish system.

#### Nitrogen and phosphorus acquisition in freshwater and brackish environments

Ammonium was the main inorganic nitrogen source observed in benthic environments (0.1–2.7 g/m^3^), but was relatively scarce in the water column (<0.04 g/m^3^), where nitrate and nitrite concentrations were also lower (Fig. 1b and Supplementary Table 4). While ammonium, derived from anthropogenic activities, can be the dominant form of N entering shallow tidal lagoon settings [54], such riverine inputs can also be nitrate-dominated [77], as observed in this study (Fig. 1b). This suggests considerable benthic regeneration of ammonium [54] in the sampled sediment (top 2 cm). Higher ammonium concentrations in the benthic zones may also be due to sedimentation of phytoplankton blooms, boosting both nitrate uptake and ammonium release in benthic environments [78, 79]. Nitrate is therefore depleted during spring bloom progression [54]. To deal with fluctuating nitrogen availability in aquatic ecosystems, microbial communities are equipped with strategies for nitrogen assimilation (e.g. fixation) and storage (cyanophycin) [80, 81]. Genes related to nitrogen fixation (*nifABDH*) were detected and expressed in non-saline and brackish sediment (Figs. 4 and 5; no transcriptomic data are available for marine sediment), although ammonium levels were around 1 g/m^3^ in some of these sites (Fig. 1b and Supplementary Table 4). Excess ammonium (>50 g/m^3^) or oxygen (>2 %) is reported to inhibit nitrogenase activity [82–84]. However, as ammonium concentrations tested were orders of magnitude higher than those we detected, ammonium concentrations in the present study may not have affected nitrogenase activity. In contrast, there were few to no *nifABDH* transcripts detected in the overlying water, likely due to low predicted iron and molybdenum concentrations [85], which is characteristic of marine water [81]. High iron and molybdenum are required for the FeMo nitrogenase cofactor in all known diazotrophs [86]. In some bacteria, excess nitrogen assimilated is stored intracellularly as cyanophycin granules comprised of nitrogen and carbon storage polymers [80, 87]. Our results indicate that genes related to cyanophycin (*cphA*) were significantly more abundant and highly expressed by *Betaproteobacteria* in non-saline water (Figs. 3a and 5, and Supplementary Figure 7), suggesting active storage and utilisation of cyanophycin granules.

Dissolved reactive phosphorus (DRP) concentrations were the highest in marine sediment pore water (0.06 g/m^3^ ± 0.03), presumably owing to the naturally higher rate of phosphorus mobilisation in marine sediments [23], and were lowest in freshwater environments (<0.01 g/m^3^, Fig. 1b). Phosphorus retention/release from sediment is complex, but is mainly controlled by increased desorption under saline conditions [88, 89]. Previous studies have shown that the high-affinity inorganic phosphate transporter *pst* system and phosphate regulon *phoB*-*phoR* (which regulates inorganic phosphate uptake) are highly upregulated under phosphate-limited conditions [90, 91]. Accordingly, data here shows that genes for phosphate uptake (*pstSABC*) and regulation (*phoU*, *phoB* and *phoR*) were negatively correlated with phosphate concentration (*ρ* = −0.49, *p* < 0.05) and were significantly more highly expressed in phosphate-limited non-saline water (Fig. 3 and Supplementary Figures 5 and 7)—primarily by *Betaproteobacteria* (*Burkholderiales*), which were highly abundant in this environment (Fig. 2a). Depending on phosphate availability, *phoR* can act as a kinase (phosphate-limited conditions) or phosphatase (phosphate-sufficient) and thereby activate or interrupt *phoB* phosphate regulation activity, respectively [91]. In phosphate-limited conditions, the phosphorylated *phoB* activates the high-affinity *pst* operon [90, 91]. Our results indicate a reliance on efficient phosphate uptake and regulation mechanisms in phosphate-limited freshwater.

#### Nitrogen cycling dynamics across the salinity gradient

Genes associated with nitrification and denitrification were predominantly expressed in brackish sediments (Fig. 5), corresponding with higher total organic carbon (TOC), total nitrogen (TN) and total recoverable phosphorus (TRP) concentrations (Fig. 1b). Results indicate that metabolic coupling between distinct taxa, to oxidise ammonia fully to nitrate, was far more common than organisms undertaking comammox along the transect (Supplementary Figure 8). We detected relatively low expression of *amoA* genes involved in comammox by the class *Nitrospira* (the genus *Nitrospira* is well known for nitrification and comammox [92]) at a single benthic freshwater site (0.9 km along the transect, Supplementary Figure 8), where nitrate was highest and ammonium concentration was lowest (Fig. 1b). In contrast, genes involved in nitrification via ammonia oxidation to nitrite (*amoA* in archaea) and nitrite oxidation (*nxrAB* in *Nitrospirae*) were highly expressed in the brackish sediments (Fig. 5 and Supplementary Figure 8). The relative abundances of ammonia-oxidising bacteria and archaea can vary in coastal and estuarine sediments, depending on the environmental conditions (salinity, oxygen and ammonium concentration) [93–95]. Although archaea constituted only a minor fraction of the overall microbiome (Fig. 2a), gene expression data indicates they were the primary ammonia oxidiser in brackish sediments and are important contributors to estuarine biogeochemical cycling.

As for nitrification, genes related to nitrous oxide reduction (*nosD*, *nosZ*), the final step of the denitrification pathway, were predominantly expressed in brackish sediments (Fig. 4) and were primarily associated with *Bacteroidetes* (Fig. 5 and Supplementary Figure 8). Results therefore suggest a strong coupling of nitrification and denitrification in brackish sediments, leading to nitrogen loss from this estuary. Steep redox gradients promote the co-occurrence of aerobic and anaerobic metabolic pathways, including the aforementioned dark carbon fixation reactions and coupled nitrification–denitrification [31, 96]. Such a gradient may be expected in brackish sediments given the combination of high mud and organic carbon contents (Fig. 1b and Supplementary Table 4), which are associated with hypoxic/anoxic conditions due to lower diffusion [97] and rapid consumption of oxygen within sediments, combined with oxygen generated by photosynthesizing diatoms at the sediment–water interface (Figs. 5 and 6).

Unlike denitrification, dissimilatory nitrate reduction to ammonium (DNRA) retains bioavailable nitrogen [98]. The first two steps in denitrification and DNRA are nitrate and nitrite reduction, of which the second step involves distinct nitrite reductase enzymes (NirKS for denitrification; NirBD/NrfA for DNRA) [99]. Results show that DNRA genes (*nrfA*, *nrfH*) were highly expressed in non-saline sediment (Fig. 4 and Supplementary Figure 8). The ratio of denitrification-*nosZ* to DNRA-*nrfA* gene expression in freshwater sediment (ratio = 0.06) was on average 20 times lower than in brackish sediment (ratio = 1.56, Supplementary Table 6), suggesting a partitioning between DNRA in freshwater sediment and denitrification in brackish sediment. Previous studies have found that a higher ratio of organic carbon (electron donor) to nitrate and a greater supply of nitrate relative to nitrite favour DNRA over denitrification [78, 100–102]. However, we did not find any relationship between carbon/nitrate or nitrate/nitrite ratios and DNRA-related gene expression in sediment (Supplementary Table 6). Although nitrate/nitrite ratios decreased across the transect (primarily due to a corresponding decrease in nitrate), nitrate concentrations were on average over ten times higher for most brackish sediment (ratios 4–34, distances ≤ 2.6 km along the transect) (Fig. 1b and Supplementary Table 4). This suggests that other unmeasured factors, such as sulfide concentrations or microbial generation times [100, 103], may instead have influenced denitrifier and DNRA activity.

#### Sulfur metabolism was most active in the brackish–marine environment

Like nitrification–denitrification, genes for sulfur metabolism, including sulfur reduction (*cysN*, *sat* and *aprA* for sulfate; *dsrABD* for sulfite) and oxidation (r*dsrAB* for sulfur), were significantly more highly expressed in brackish sediments (Fig. 3a, Supplementary Figures 9-10). Expression was concomitant with highly abundant *Gammaproteobacteria* and *Deltaproteobacteria* capable of oxidising sulfur and reducing sulfate, respectively (Figs. 2a and 5). This is consistent with greater sulfide accumulation in brackish sediments owing to high mud and limited oxygen diffusion [30]. Taken together, these results reinforce the findings of studies indicating the brackish environment is a hotspot for biogeochemical cycling [104, 105]. However, the expression of thiosulfate oxidation genes (*soxBCY*) by *Alphaproteobacteria* was significantly higher in marine water (LDA score ≥ 2, Fig. 3a and Supplementary Figure 9) and positively correlated with high sulfate concentrations typical of marine water (*ρ* = 0.76, *p* < 0.05; Fig. 1b and Supplementary Figure 5). Thiosulfate can serve as an electron donor for anoxygenic photosynthesis [106] and be oxidised to sulfate via the Sox pathway [107]. Bacterial groups that perform aerobic anoxygenic photosynthesis (AAP), including alpha-, beta- and gamma-proteobacteria, are widely distributed in the surface ocean [108, 109]. Greater expression of genes related to thiosulfate oxidation (Supplementary Figure 9) and anoxygenic photosynthesis (*pufLM*) by planktonic marine *Alphaproteobacteria* (Figs. 5 and 6) suggests these bacteria may have coupled thiosulfate oxidation with photoheterotrophy in relatively carbon-limited marine water (DNPOC 1.7 versus 2.2 g/m^3^ in brackish water) [110].

## Conclusions

Across the river–marine transect, in a tidal lagoon setting, the microbial community and diversity were dominated by bacteria. Contrasting diversity trends were observed between the water column and sediments, and between prokaryotes and microeukaryotes, reflecting the strong geochemical differences between these environments, and the distinct physiologies and ecosystem roles of these taxa groups. While archaea and microeukaryotes represented only a minor fraction of the overall communities, they played significant roles in ammonia oxidation and photosynthesis, respectively. Microorganisms employed distinct osmoregulation strategies between freshwater and saline habitats by actively expressing diverse osmoregulation genes encoding potassium, sodium and glycine betaine transporters across the transect. Results indicate a significant predominance of phosphate acquisition in non-saline water, along with microeukaryotic-driven primary production, and prokaryotic nitrification–denitrification and sulfur metabolism in the brackish sediments, corresponding with large differences in resource availability. Overall, our study demonstrates that differences in salinity and nutrients impose significant biological boundaries between sediment and water, and among non-saline, brackish and marine environments.

## Materials and methods

### Sampling strategy

Sampling was undertaken on 12 November 2018 within a low tide period. Nine sites (sites 1–9, Fig. 1a; site photos, Supplementary Figure 11) were selected covering a freshwater-to-marine salinity (*S*) gradient (0.2 < *S* < 35) across a 5-km-long transect of the Waiwera river and estuary, north of Auckland, New Zealand (36° 33′ 02.4″ S, 174° 39′ 08.1″ E). For molecular analyses, at least 10 L of water was collected between 0.2 and 1 m below the surface of all sites and sequentially filtered through sterile 1.2-μm and 0.22-μm mixed cellulose ester filters (Merck Millipore, MA, USA). Triplicate water samples (unfiltered) were similarly collected in polyethylene bottles for chemical analysis at Hill Laboratories (Hamilton, New Zealand). The top 2 cm of sediment was collected at three locations approximately 5 m apart at each site and wet sieved through a 1-mm mesh sieve to remove stones, plant material and macrofauna. All filter and sediment samples for molecular analyses were immediately preserved with LifeGuard Soil Preservation Solution (Qiagen, MD, USA) in sterile 50-mL Falcon tubes (1:3 wet sediment-to-LifeGuard ratio, individual water samples were filtered and preserved within 5–10 min of sample collection) and stored at −80 °C until extraction. Samples for mud content and chemical analyses were stored at 4 °C.

### Mud content measurement

Sediment samples were oven dried at 105 °C for 12 h prior to sieving. Sediments were serially sieved using mesh sizes ranging from 1 mm to 63 μm with a sieve shaker. Fractions retained on each mesh were weighed to determine mud and sand contents. Mud (clay and silt) and sand are identified as sediment with sizes of <63 μm and >63 μm, respectively [111]. Mud content was calculated as follows: the weight of mud fraction/weight of sediments from all fractions × 100 %.

### Physiochemical and nutrient analyses

Temperature, pH and salinity were measured on-site using a Pocket Pro+ Multi 2 Tester (HACH, CO, USA). All sediment and water samples were sent to Hill Laboratories (Hamilton, New Zealand) for processing. All nutrients were measured according to standard American Public Health Association (APHA) protocols [112]. TRP, TS, TN and TOC were only measured from sediment samples. All other nutrients were measured from water column and sediment pore water samples. Briefly, sediment TOC, following pre-treatment with acid to remove carbonates, was analysed using an Elementar Vario MAX Combustion Analyser (Langenselbold, Germany) with catalytic combustion (at 900 °C) and TN was measured via an Lachat Quikchem Series 2 Flow injection Analyser (Lachat Instruments, CO, USA). Total sulfur was measured using a LECO SC-32 Sulfur Determinator (MI, USA), and TRP was analysed using a Aquakem Konelab 600 Discrete Analyser (Thermo Fisher Scientific, MA, USA). Water samples were prefiltered through 0.45-μm membrane filters and saline samples diluted prior to analyses. Total ammonical-N, nitrate, nitrite and DRP were measured via a Lachat Quikchem Series 2 flow injection analyser (Lachat Instruments, CO, USA). Sulfate was analysed using a Dionex Ion Chromatography system (Sunnyvale, CA, USA). Samples for DNPOC were acidified to remove inorganic C before addition of persulfate, heating to a temperature of above 375 °C and analysis via a Sievers Innovox TOC analyser (SUEZ Analytical Instruments, CO, USA). River turbidity data, collected by Auckland Council at GPS coordinates 36° 33′ 01.4″ S 174° 39′ 59.3″ E, were obtained from the Land, Air, Water Aotearoa (https://www.lawa.org.nz/).

### Nucleic acid extraction and sequencing

Both RNA and DNA were co-extracted using RNeasy PowerSoil Total RNA Kits and RNeasy PowerSoil DNA Elution Kits (Qiagen, MD, USA). Total RNA and DNA from each sample were isolated from 2–8 g of sediment or 1–5 g of filter (1/3-1 filter, 10L water per filter) with collected planktonic biomass (both 1.2 μm and 0.22 μm combined). Extracted nucleic acids were stored at −80 °C. RNA samples were treated with DNaseI. DNA removal was verified by PCR amplification of the 16S rRNA genes using 515F/806R primers [113, 114], JumpStart REDTaq Readymix (Sigma-Aldrich, MO, USA) and 2 μL of the template (sample or positive control), under the following conditions: 95 °C for 5 min; 55 cycles: 95 °C for 45 s, 50 °C for 60 s, 72 °C for 90 s; 72 °C for 10 min. Visualisation by gel electrophoresis was used to confirm no amplification in DNase-treated samples. RNA was purified using RNA Clean & Concentrator-5 Kits (Zymo Research, CA, USA). DNA extracts were further purified by mixing with nuclease-free 0.3 M sodium acetate (final concentration; Sigma-Aldrich, MO, USA) and 0.1 μg/μL glycogen (final concentration) and re-precipitating with 2:1 volumes of cold ethanol following incubation for 30 min at −20 °C. Pellets were washed with 70% ethanol and re-eluted in nuclease-free water. High molecular weight DNA was confirmed via gel electrophoresis. DNA and RNA concentrations and quality were further assessed using a Nanodrop 2000c Spectrophotometer (Thermo Fisher Scientific, MA, USA) and also, for RNA, an Agilent 2100 Bioanalyzer (Agilent Technologies, CA, USA) using the Agilent RNA 6000 Nano Kit. Low-quality RNA extracts (without obvious 16S/23S peaks in bioanalyzer electropherogram) from sandy sediment at marine sites 8 and 9 were excluded from further analysis. The remaining 36 DNA and 30 RNA extracts were prepared for metagenomic and metatranscriptomic sequencing. Prior to RNA library preparation, ribosomal RNAs were depleted using the Ovation Universal Prokaryotic RNASeq, Prokaryotic AnyDeplete (TECAN, Zürich, Switzerland) system, according to the manufacturer’s instructions. Library preparation and sequencing were undertaken by the Otago Genomics Facility (University of Otago, Dunedin, New Zealand). Metagenome libraries were prepared using the Illumina TruSeq DNA Nano kit (with 550-bp insert sizes). Metatranscriptome libraries were prepared using the Ovation Complete Prokaryotic RNASeq kit. Paired-end 2 × 250 bp sequencing for DNA (over 14 lanes) and 2 × 125 bp sequencing for RNA (over 14 lanes) were performed using the Illumina HiSeq 2500 platform with V2 (DNA) and V4 (RNA) chemistry. All sequence data have been deposited with NCBI under BioProject PRJNA668816.

### Sequence read processing

Ribosomal RNA reads were first removed from metatranscriptomes using SortMeRNA v2.1 [115] with bacterial, archaeal and eukaryotic rRNA databases, and results were passed to repair.sh (BBMap [116]) to regenerate paired-end reads. Metagenomic and non-ribosomal metatranscriptomic read quality was inspected using FastQC [117]. Reads were trimmed using Trimmomatic v0.38 [118] with a minimum Phred score of 30 and adapter sequence removal via the ILLUMINACLIP option with Illumina universal adapter sequence (AGATCGGAAGAG). Reads were also trimmed to a maximum of 240 bp (DNA) and 115 bp (RNA) long to remove additional low-quality bases, and reads <80 bp (DNA) and <60 bp (RNA) long were discarded. A sediment sample from site 9, replicate 1 (S9R1), was subjected to additional trimming (headcrop 10) due to low base quality.

### Small subunit (SSU) rRNA gene reconstruction

Near full-length 16S and 18S rRNA gene sequences were reconstructed from each metagenome, over 50 iterations and using a joining threshold of 97%, with EMIRGE [119] and the SILVA SSU Ref NR 99 138 database. We detected and removed 2670 chimeras with the uchime_ref command [120]. Sequences from across samples were concatenated and clustered into OTUs (97% threshold) with UCLUST [120], then rarefied to 3343 sequences per sample, resulting in a total of 7334 unique OTUs with a minimum count across samples ≥2 (Supplementary Table 7). SSU rRNA gene relative abundances were based on normalised priors from the final EMIRGE iteration.

### Metagenome assembly, taxonomic and functional annotations

Trimmed reads from individual water samples or from spatial triplicate sediment samples from the same site were assembled or co-assembled, respectively, using metaSPAdes [121] with k-mer values: 41, 61, 81, 101 and 127, resulting in 143 million contigs (Supplementary Figure 12). A k-mer-based profiler, Kraken2 v2.1.2 [122], was used to assign taxonomy to the trimmed reads based on compositional similarity to publicly available genome databases from RefSeq [123]. Species relative abundances were further estimated using a Bayesian approach with Bracken v2.6.0 [124]. Data were rarefied to 2,543,737 reads per sample, and species belonged to *Streptophyta* and *Chordata* were removed, resulting in a total of 5136 unique species (Supplementary Table 8). Open reading frames (ORFs) were predicted and translated into protein sequences using Prodigal v2.6.3 [125]. Protein sequences were searched against a library of hidden Markov models (HMMs) consisting of TIGRFAMs [126], Pfam [127] and custom HMMs for metabolic pathways using HMMER3 [128]. Marker genes for the pathway of interest and the cut-off values for HMM scores were derived from Anantharaman et al. [129], Pfam [127], TIGRFAM [126] and FunGene [130] databases (Supplementary Table 9). Phylogenetic analyses were conducted on concatenated *dsrAB* genes to further classify them into *dsrAB* (in sulfate/sulfite-reducing bacteria) and *rdsrAB* (in sulfur-oxidising bacteria) using FastTree v2.1.10 [131]. Reference protein sequences for phylogenetic analyses were obtained by BLAST searches against the NCBI nr protein database [132]. Taxonomic affiliations of functionally annotated genes were assigned with DIAMOND BLASTP [133] with default settings against the NCBI nr protein database (downloaded on 16 February 2020), using a weighted lowest common ancestor (LCA) approach. We retained only hits with at least 70% query coverage, 80% identity and bit scores within 5% of the top hit for LCA identification.

### Metagenome and metatranscriptome read mapping and counts

DNA and RNA (non-rRNA) reads were mapped back to metagenomic contigs using BBMap v37.93 [116] with default parameters. Read counts were calculated for each predicted open reading frame (ORF) using featureCounts v2.0.0 [134]. Gene and transcript counts were normalised using gene or transcript per million mapped reads (TPM) to measure the bulk contribution of the community [135, 136]. TPM = (number of reads mapped to the gene/gene length)/sum (number of reads mapped to the gene/gene length) × 1,000,000.

### Statistical analyses

Unless otherwise stated, the following statistical analyses were carried out in R environment version 3.5.1 [137]. Shannon–Wiener indices, Simpson’s indices and relative OTU richness were determined using the vegan package [138] and the rarefied OTU table combining reconstructed 16S and 18S rRNA genes. A Wilcoxon signed-rank test was performed to determine significant differences in observed diversity between the water column and sediment. Distance–decay relationships for microbial communities and functional gene groups were constructed by first calculating pairwise Bray–Curtis dissimilarities among sites using the vegdist function in the vegan package [138], and fitting negative exponential functions via a log-linked generalised linear model (GLM) or local polynomial regression (LOESS) model. NMDS ordinations were plotted based on Bray–Curtis dissimilarities, and environmental variables were fitted with 999 Monte Carlo permutation tests using the vegan package [138]. Pairwise permutational multivariate analyses of variance (PERMANOVA) with Benjamini–Hochberg corrections were carried out to test for statistically significant variance among multivariate microbial community data using the pairwiseAdonis package [139]. Spearman’s correlation coefficients between the relative abundances of specific taxa and environmental parameters were calculated using the R cor function. Taxa with *p* < 0.05 were determined to be significantly correlated with the environmental parameters. The bioenv function in R’s vegan package [138] was used with Spearman’s rank correlations to search over subsets of the continuous environmental variables to determine the best explanatory variables for community composition. A multivariate regression tree was plotted using mvpart package [140], with 1000 cross-validations and tree size = 8 to identify the main predictors that correlate with microbial community structure. As both salinity and distance were highly correlated and exhibited multicollinearity, we used salinity (and excluded distance) as one of the predictor variables for the analysis. The decision tree identifies variance in microbial community composition (Bray–Curtis dissimilarity) caused by threshold values of key site physicochemical attributes. The values attached to each branch of the tree mark the physicochemical criteria used by the regression tree to group samples based on differences in community composition.

The correlation between gene and transcript relative abundances was measured using the R cor.test function. Mantel tests were run to assess Spearman’s correlations between functional and taxonomic community dissimilarity matrices based on Bray–Curtis dissimilarities. LEfSe was applied to metagenomic and metatranscriptomics data to identify environment-associated metabolic functions by utilising Kruskal–Wallis and Wilcoxon rank-sum tests to determine variations occurring between and within environments with a linear discriminant analysis (LDA) score of ≥2 [141]. To determine co-expressed genes associated with environmental parameters, weighted correlation network analysis was also performed using transcript counts normalised by TPM as input data to find clusters of highly correlated gene transcripts by using WGCNA package [142] with minimum module size = 5 and soft-thresholding power = 14 (*R*^2^ = 0.81) to relate the functions or modules to one another and to environmental parameters.

## Supplementary Information

**Additional file 1.** Supplementary tables.

**Additional file 2.** Supplementary results and discussion, and figures.

## Data Availability

The data generated in the current study are all publicly available. All sequence data have been deposited with NCBI under BioProject PRJNA668816. R scripts for plotting figures and statistical analyses are available at https://github.com/HweeSze/Waiwera_manuscript_plots.
