# Supplementary material for: Microbial river-to-sea continuum: gradients in benthic and planktonic diversity, osmoregulation and nutrient cycling
Source: Microbiome. 2021 Sep 20;9:190. doi: 10.1186/s40168-021-01145-3 (PMC8454136; doi:10.1186/s40168-021-01145-3)
Supplement: Supplementary file 3 — Additional file 2. Supplementary results and discussion, and figures. [file 40168_2021_1145_MOESM3_ESM.pdf]

Supplementary Information

Title: Microbial river-to-sea continuum: gradients in benthic and planktonic diversity, osmoregulation and nutrient cycling

Authors: Hwee Sze Tee[1a]; David Waite[1Ŧb]; Gavin Lear[1c]; Kim Marie Handley[1d*]

[1]School of Biological Sciences, University of Auckland, Auckland,1010 New Zealand

[Ŧ]Current address: Ministry for Primary Industries, Auckland, New Zealand

Results and Discussion

Spatial community structuring across the river to marine transect. EMIRGE-reconstructed SSU rRNA analysis showed *Burkholderiales*, *Micrococcales* and *Flavobacteriales* were ubiquitous in the water column, although the relative abundance of *Burkholderiales* and *Micrococcales* were negatively correlated with salinity. Results also indicate that *Cytophagales* (16.2 % ± 2.4) dominated the freshwater planktonic environment, whereas *Rhodobacterales* (9.6 % ± 4.9), *Oceanospirillales* (8.4 % ± 6.4) and SAR11 clade (10.3 % ± 5.7) were prevalent in brackish and marine water (Fig. 2a). We also observed a large peak in eukaryotic phytoplankton comprising phylum *Cryptophyta* (genus *Hemiselmis*, 5.0 % relative abundance) in the mid-brackish site 5 (salinity: 24). Phytoplankton spring blooms are often associated with a rise in surface temperature, light intensity and dissolved nutrients [1,2]. Previous studies suggest *Cryptomonad* blooms can be initiated by higher dissolved nutrient concentrations that occur during spring due to precipitation and greater mixing in the water column [3], which is when our sampling was conducted. Among sediments environments, the majority of sequences recovered from the freshwater sediments belonged to *Burkholderiales* (7.1 % ± 1.5), and *Rhizobiales* (4.6 % ± 0.4), whereby *Rhodobacterales* (4.0 % ± 0.9), *Cellvibrionales* (2.2 % ± 0.9), *Desulfobulbales* (3.4 % ± 1.7), and *Actinomarinales* (1.9 % ± 1.2) were mainly found in brackish and marine sediments. Although archaea constituted only a minor fraction of the overall community (Fig. 2a), we observed that the archaeal ammonia-oxidizers and methane-producer were found widespread in benthic environments, suggesting their importance in biogeochemical cycling in these environments [4,5]. The methane-producing archaea, including *Methanosarciniales* and *Methanomicrobiales* from phylum *Halobacterota* were observed in freshwater-brackish sediments, whereas the ammonia-oxidizer *Nitrososphaerales* and *Nitrosopumilales* from phyla

Crenarchaeota (the most prominent archaeal phylum) were found abundant in freshwater-brackish sediments and brackish-marine sediments respectively.

Taxonomic classification and estimated abundances based on metagenomic sequence data

One of the well-known bias in marker gene studies is the variation in gene copy number per bacterial and eukaryal genomes [6–8]. Therefore, to validate the SSU rRNA relative abundance profile, we used Kraken2 v2.1.1 [9] to classify metagenomic reads into species, then estimated the species-level sequence abundances using a Bayesian approach with Bracken v2.6.0 [10]. On average, 9.9 % ± 3.5 of metagenomic reads were taxonomically classified to their lowest common ancestor, resulting in a total of 5,197 unique species. In agreement with previous works that showed similar microbial abundance distributions from both amplicon and metagenomic-inferred species abundance[11,12], both EMIRGE (assembled 16S SSU rRNA gene) and Kraken/Bracken derived community distributions exhibited similar trends across the salinity gradient (Spearman's correlation coefficient ρ=0.91), with a clear separation between water and sediment community composition. *Burkholderiales*, *Micrococcales* and *Rhizobiales* dominated the non-saline environment, whereas *Rhodobacterales, Pelagibacterales* and *Flavobacteriales* were more abundant in saline environment (Supplementary Fig. 1). Similar to the SSU rRNA analysis, the Kraken2 approach indicated an increase in the relative abundances of photoautotrophs in the brackish water, in particular phylum *Cryptophyta* (order *Pyrenomonadales*). Both Kraken/Bracken and EMIRGE results showed that archaea comprised <1% of the water communities and <1.5% of sediment communities, and that *Nitrosopumilales*, and to a lesser extent *Methanosarciniales*, were relatively abundant (with *Nitrosopumilales* being particularly abundant in saline sediments). However, a greater diversity of archaea was identified by EMIRGE.

Correlations between co-expressed gene clusters and environmental factors

A weighted gene co-expression network analysis (WGCNA) was conducted to determine gene expression patterns and interactions across the transect. Results revealed seven major co-abundance clusters that were arbitrarily assigned colors grey, yellow, green, red, brown, turquoise, and blue (Supplementary Fig. 5). The grey cluster shows genes that were unassigned due to absence of co-expression with other genes. In agreement with LEfSe analysis, genes in the blue cluster (related to potassium transporter kdp, complete ammonia oxidation,

reverse TCA cycle and DNRA) were negatively correlated with salinity (ρ=0.78, p<0.01) and were found highly expressed in non-saline sediments, whereas the highest eigenvalues of the brown cluster, containing genes related to cyanophycin and phosphate acquisition, was found in non-saline water. Turquoise (nitrogen and sulfur metabolism) and green (primary production) clusters showed higher expression in the brackish environments and were positively correlated with salinity (ρ=0.78-0.81, p<0.01; Supplementary Fig. 5), suggesting that brackish environments act as a microbial hotspot for biomass generation and activity [13–15]. Genes related to thiosulfate oxidation and anoxygenic photosynthesis within red cluster were found highly expressed in marine water, whereas yellow cluster, comprising genes related to glycine betaine transporter, Na^+/H^+ antiporter, and potassium transporter Trk, were found highly expressed in brackish sediments and marine water. These findings support LEfSe results and emphasise the strong division of metabolisms across the salinity gradient in water and sediment environments, which may be largely attributed to differences in lineage adaptation to salinity [16,17] and nutrient availability [18–20].

References

1. Lewandowska AM, Striebel M, Feudel U, Hillebrand H, Sommer U. The importance of phytoplankton trait variability in spring bloom formation. ICES J Mar Sci. 2015;72:1908–15.

2. Winder M, Cloern JE. The annual cycles of phytoplankton biomass. Philos Trans R Soc B Biol Sci. 2010;365:3215–26.

3. Egerton T, Morse R, Marshall H, Mulholland M. Emergence of algal blooms: The effects of short-term variability in water quality on phytoplankton abundance, diversity, and community composition in a tidal estuary. Microorganisms. 2014;2:33–57.

4. French E, Kozlowski JA, Mukherjee M, Bullerjahn G, Bollmann A. Ecophysiological characterization of ammonia-oxidizing archaea and bacteria from freshwater. Appl Environ Microbiol. American Society for Microbiology; 2012;78:5773–80.

5. Pajares S, Ramos R. Processes and microorganisms involved in the marine nitrogen cycle: knowledge and gaps. Front Mar Sci. Frontiers; 2019;6:739.

6. Louca S, Doebeli M, Parfrey LW. Correcting for 16S rRNA gene copy numbers in microbiome surveys remains an unsolved problem. Microbiome. 2018;6:41.

7. Lavrinienko A, Jernfors T, Koskimäki JJ, Pirttilä AM, Watts PC. Does intraspecific variation in rDNA copy number affect analysis of microbial communities? Trends Microbiol. Elsevier; 2021;29:19–27.

8. Torres-Machorro AL, Hernández R, Cevallos AM, López-Villaseñor I. Ribosomal RNA genes in eukaryotic microorganisms: witnesses of phylogeny? FEMS Microbiol Rev. 2010;34:59–86.

9. Wood DE, Lu J, Langmead B. Improved metagenomic analysis with Kraken 2. Genome Biol. 2019;20:257.

10. Lu J, Breitwieser FP, Thielen P, Salzberg SL. Bracken: estimating species abundance in metagenomics data. PeerJ Comput Sci. PeerJ Inc.; 2017;3:e104.

11. Durazzi F, Sala C, Castellani G, Manfreda G, Remondini D, De Cesare A. Comparison between 16S rRNA and shotgun sequencing data for the taxonomic characterization of the gut microbiota. Sci Rep. 2021;11:3030.

12. Rausch P, Rühlemann M, Hermes BM, Doms S, Dagan T, Dierking K, et al. Comparative analysis of amplicon and metagenomic sequencing methods reveals key features in the evolution of animal metaorganisms. Microbiome. 2019;7:133.

13. Andersson A, Brugel S, Paczkowska J, Rowe OF, Figueroa D, Kratzer S, et al. Influence of allochthonous dissolved organic matter on pelagic basal production in a northerly estuary. Estuar Coast Shelf Sci. 2018;204:225–35.

14. Hargrave BT, Holmer M, Newcombe CP. Towards a classification of organic enrichment in marine sediments based on biogeochemical indicators. Mar Pollut Bull. 2008;56:810–24.

15. Cloern JE, Foster SQ, Kleckner AE. Phytoplankton primary production in the world's estuarine-coastal ecosystems. Biogeosciences. 2014;11:2477–501.

16. Walsh DA, Lafontaine J, Grossart H-P. On the eco-evolutionary relationships of fresh and salt water bacteria and the role of gene transfer in their adaptation. In: Gophna U, editor. Lateral Gene Transf Evol. New York, NY: Springer; 2013. p. 55–77.

17. Henson MW, Lanclos VC, Faircloth BC, Thrash JC. Cultivation and genomics of the first freshwater SAR11 (LD12) isolate. ISME J. Nature Publishing Group; 2018;12:1846–60.

18. Allan JD, Ibañez Castillo MM. Stream ecology: structure and function of running waters. 2nd ed. Dordrecht, Netherlands: Springer; 2007.

19. Heinrichs ME, Mori C, Dlugosch L. Complex interactions between aquatic organisms and their chemical environment elucidated from different perspectives. In: Jungblut S, Liebich V, Bode-Dalby M, editors. YOUMARES 9 - Oceans Our Res Our Future. Cham: Springer; 2020. p. 279–97.

20. Cloern JE, Jassby AD, Schraga TS, Nejad E, Martin C. Ecosystem variability along the estuarine salinity gradient: Examples from long-term study of San Francisco Bay. Limnol Oceanogr. 2017;62:272–91.

Supplementary Figures

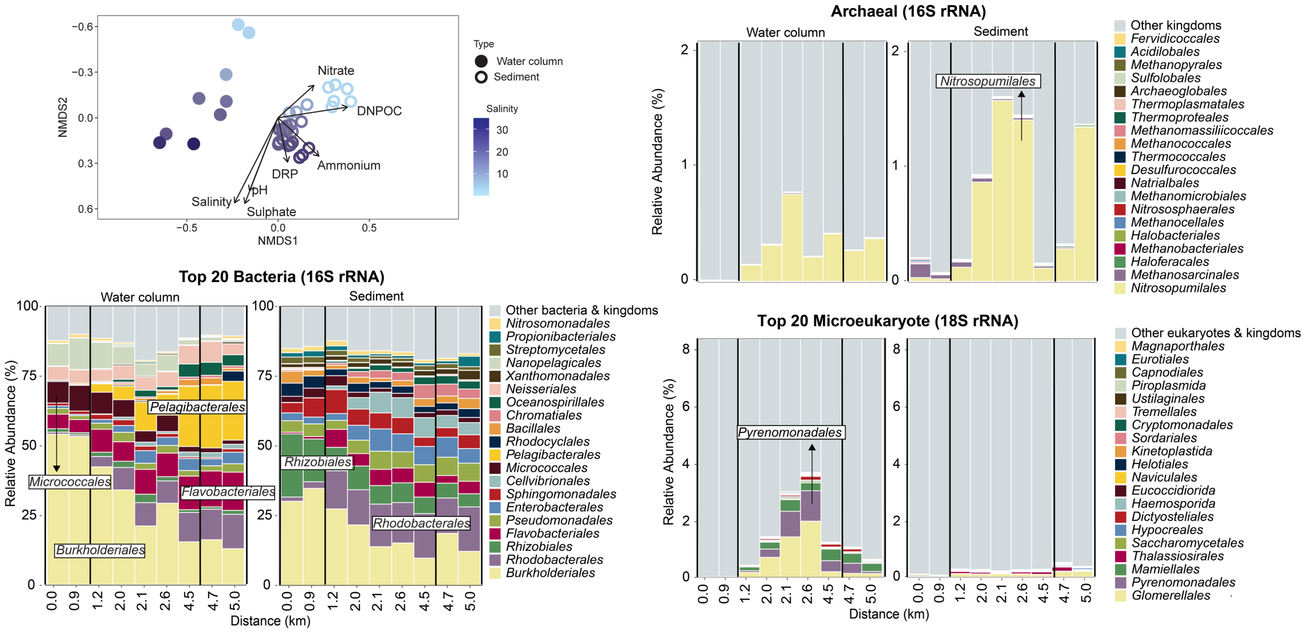

Supplementary Figure 1. NMDS ordination of Kraken-derived species based on Bray-Curtis dissimilarities and community distribution. Samples for NMDS analysis are coloured according to salinity. Environmental vectors were fitted onto the NMDS scores of the microbial community by the R-function envfit ($p<0.05$, permutation = 999). Bar plots indicates Bracken2-estimated abundances of metagenomic-derived species. Taxa are coloured at order level. For each bar plot, samples from the water column (left) and sediment (right), are further categorized into freshwater, brackish or marine, as indicated in the top left plot.

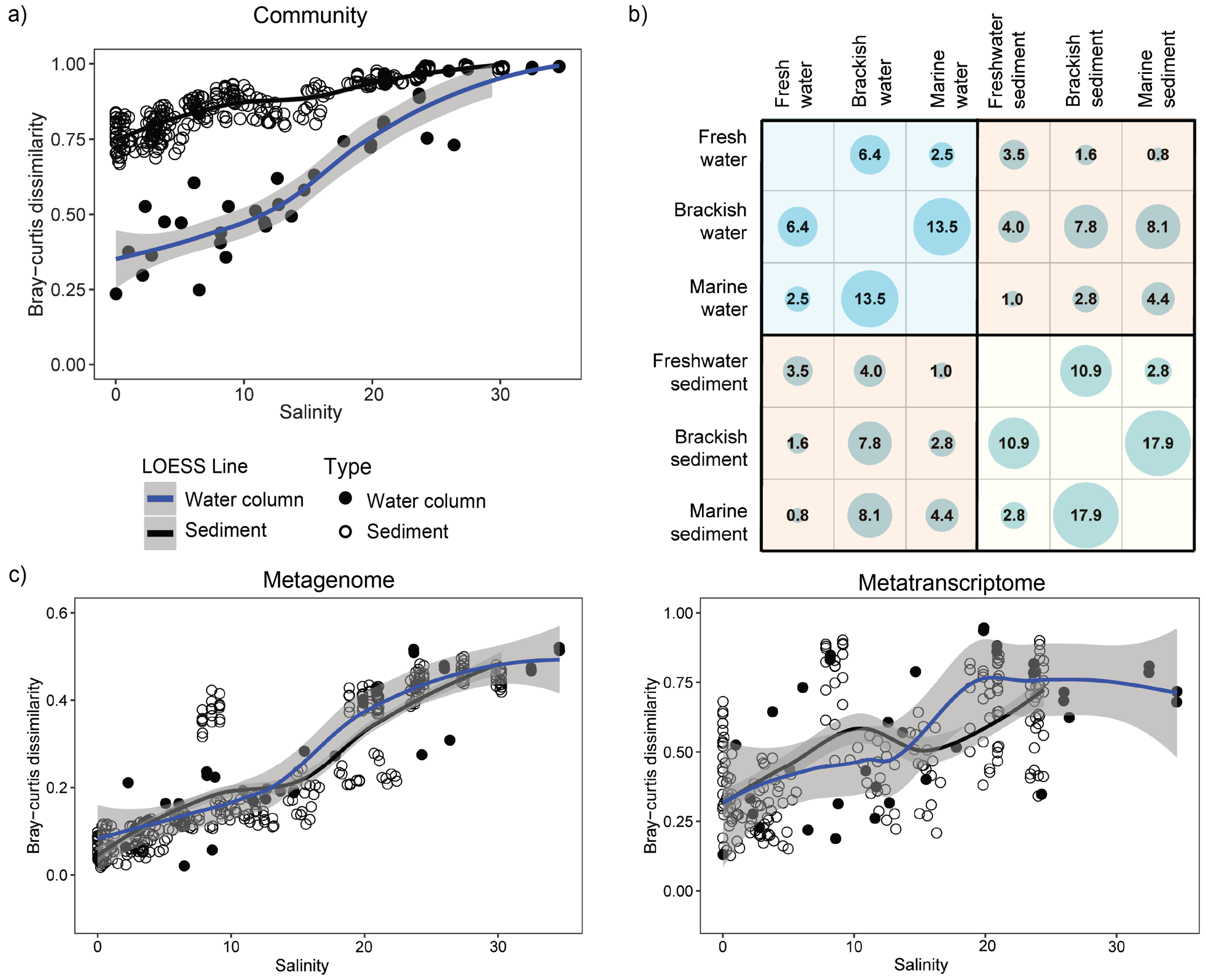

Supplementary Figure 2. Plots showing community composition dissimilarity and taxa shared across the aquatic transect. a) Distance-decay relationship of community dissimilarity (Bray–Curtis index) with respect to salinity. The regression lines are fitted using a LOESS model: water column (lower blue line), sediment (upper black line). Each data point represents a pairwise comparison of samples from either the water column (closed circle) or sediment (open circle). b) Percentage of shared taxa across six distinct habitats. The size of circles and text within circles represent the percentage of shared OTUs between two habitats. The background colour indicates the pairwise comparison group (blue = water vs water, orange = water vs sediment, yellow = sediment vs sediment). c) Distance-decay relationship of functional dissimilarity based on genes and transcripts related to nutrient acquisition and osmoregulation.

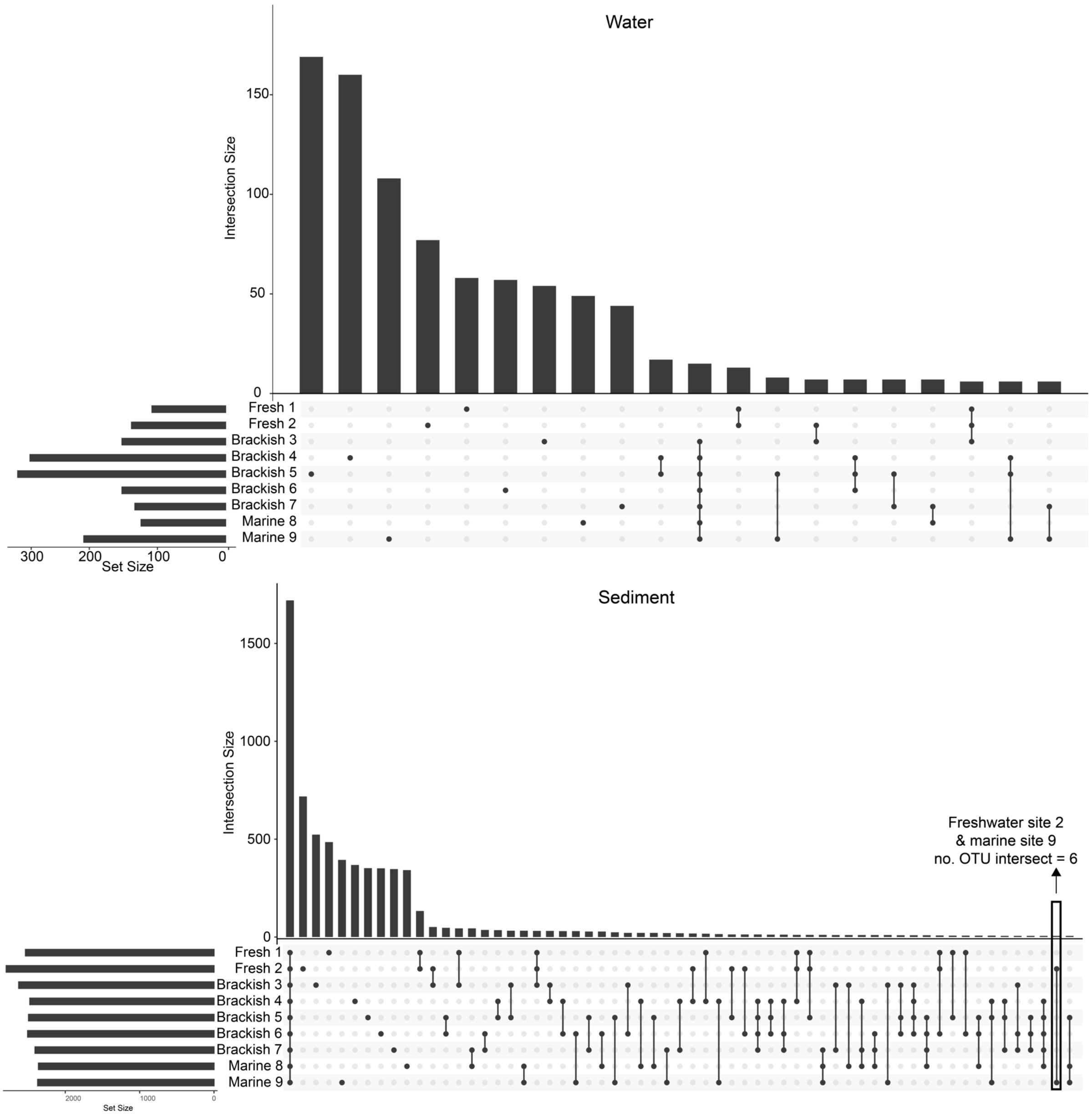

Supplementary Figure 3. Number of OTUs shared across nine water (top) and sediment (bottom) sites. The vertical bar plots indicate the number of OTUs exclusive to one water or sediment site (individual dot) or shared between the intersecting sites (connecting dots). The horizontal bar plots indicate the total number of OTUs per sample (relative species richness). Sites or intersecting sets with OTUs >6 are shown in the plot.

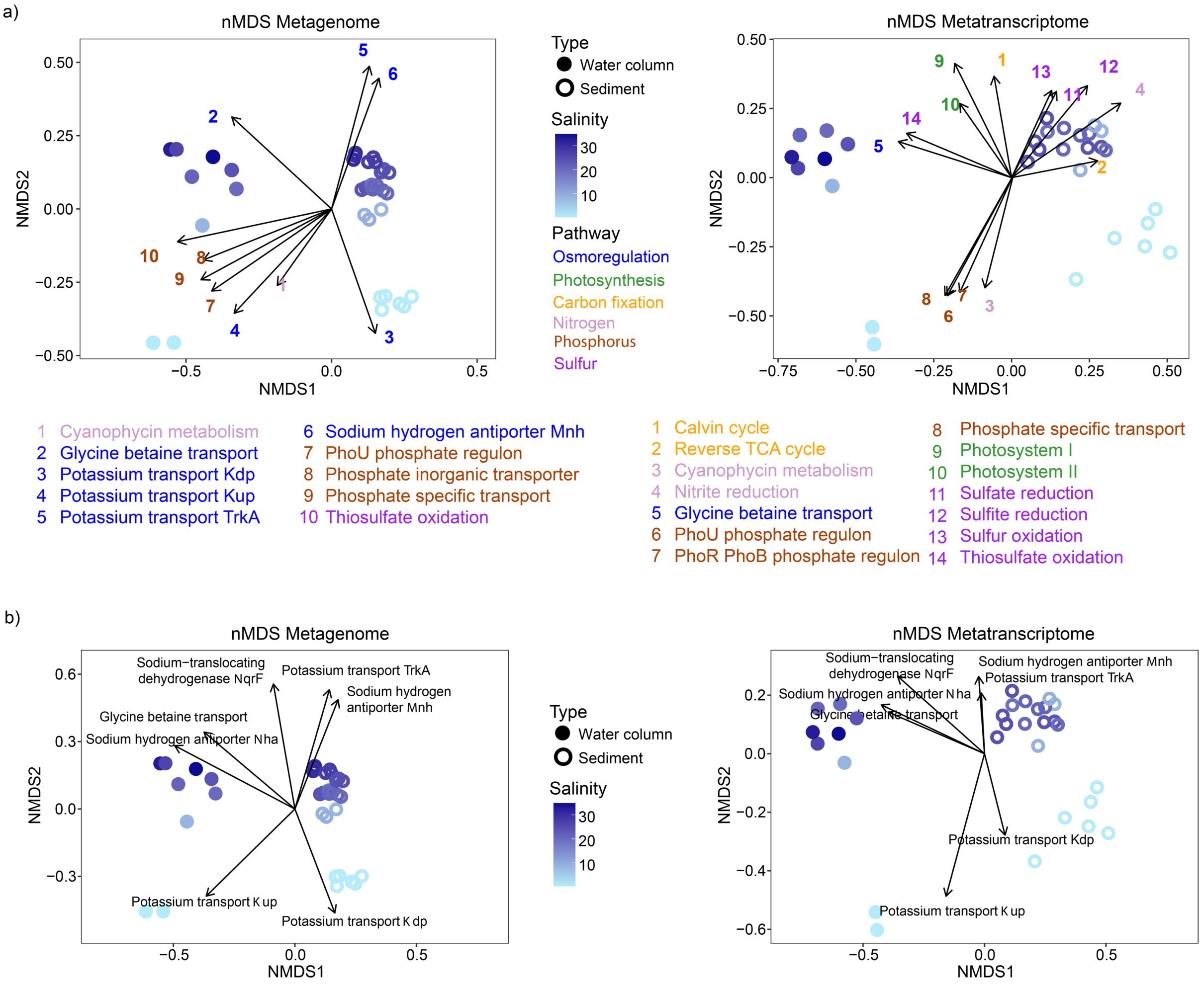

Supplementary Figure 4. NMDS ordination of functional genes/gene transcripts. The underlying data were generated using functional gene/transcript abundances related to nutrient acquisition and osmoregulation based on Bray-Curtis dissimilarities. Samples are coloured according to salinity for both the water column (closed circles) and sediment (open circles). Fitted black vectors ($p<0.05$, permutation = 999) in a) indicate key functional pathways identified by LEfSe; numbers at arrow tips indicate gene functions, and number colour denotes pathway categories (key, upper middle). Black vectors in b) indicate fitted functions related to osmoregulation.

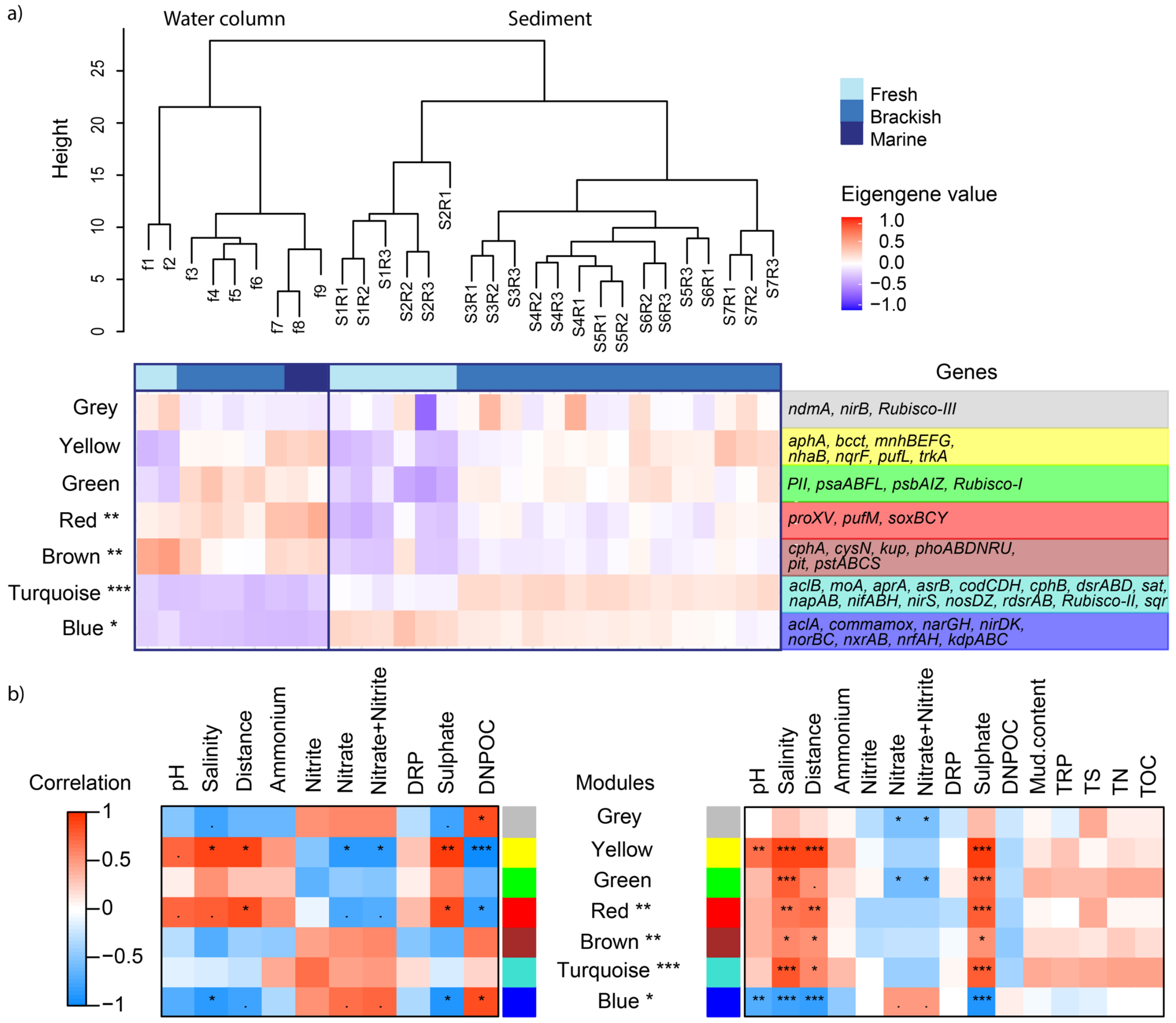

Supplementary Figure 5. Plots showing weighted gene co-expression network analysis (WGCNA) and Spearman's correlations of gene expression and environmental data. a) Unsupervised hierarchical cluster analysis showing the relationships among 9 water and 21 sediment transcriptomic samples, and heatmap showing the gene cluster (or module) eigengene values. The expression level of a cluster eigengene is defined as the first principal component of a given module and used to represent the overall expression level of a cluster. b) Heatmaps showing Spearman's correlations between the cluster eigenvalue and the environmental factors for water (left plot) and sediment (right plot) with 5 additional parameters (mud content to TOC) specific to sediment. Significant *p*-values are shown as follow: ***≤ 0.001; **≤ 0.01; *≤ 0.05; .≤ 0.1.

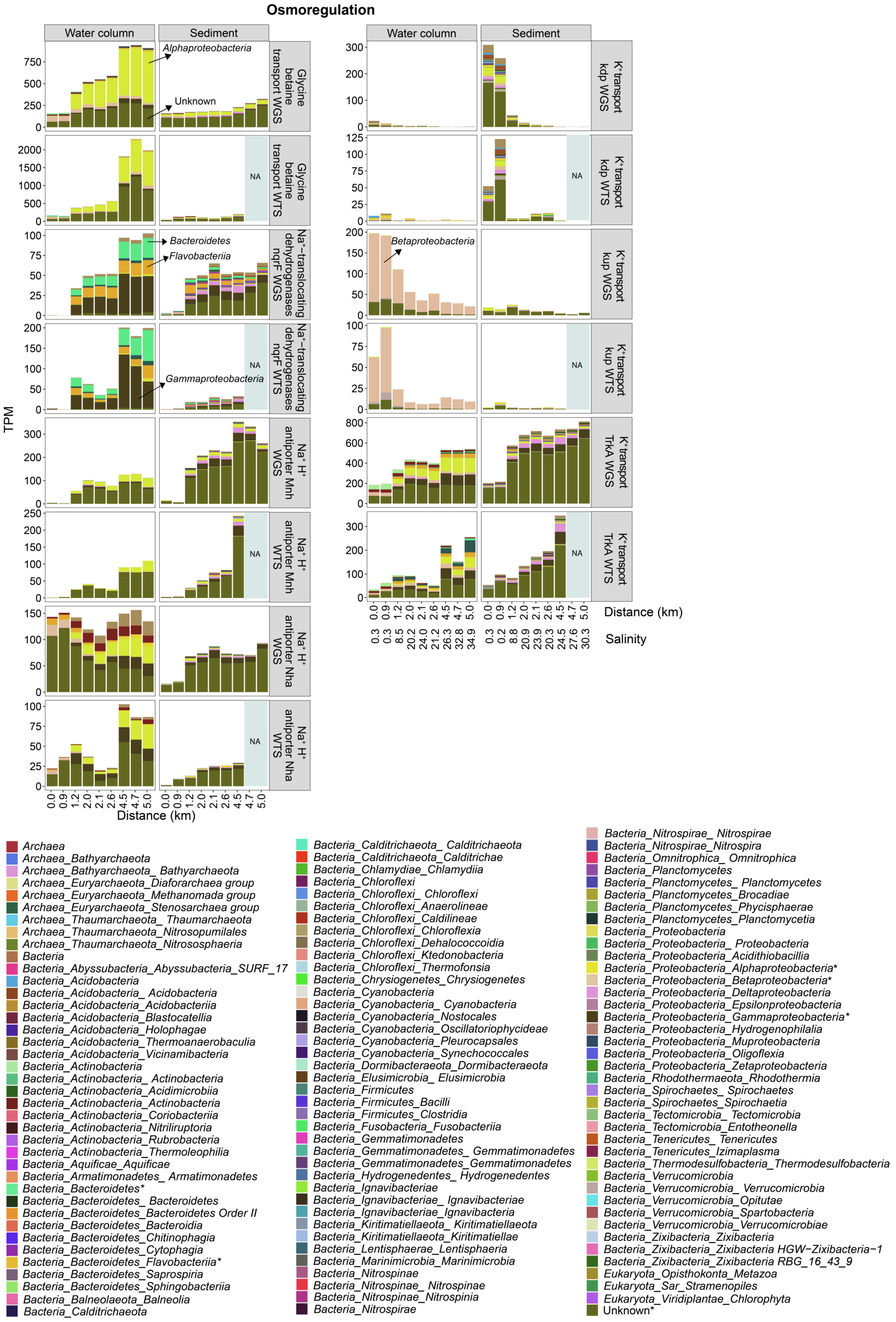

Supplementary Figure 6. Stacked bar plot showing the distribution of taxon-specific genes and transcripts (genes/transcripts per million mapped reads, TPM) involved in **osmoregulation** across the study sites. NA indicates that no transcriptomic data are available for marine sediments. Abbreviations: metagenome (WGS), metatranscriptome (WTS).

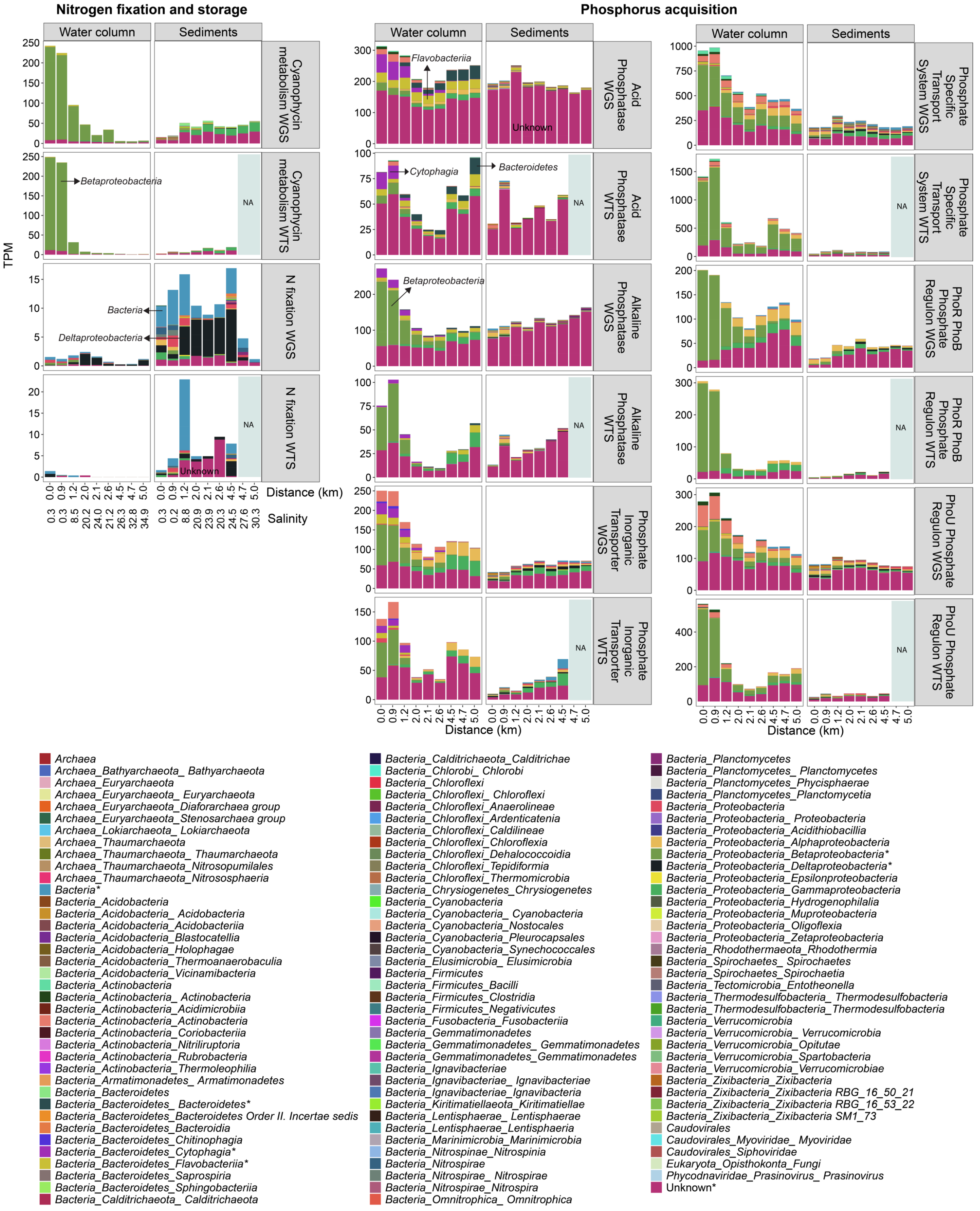

Supplementary Figure 7. Stacked bar plot showing the distribution of taxon-specific genes and transcripts (genes/transcripts per million mapped reads, TPM) involved in **nitrogen fixation and storage** and **phosphorus acquisition** across the study sites. NA indicates that no transcriptomic data are available for marine sediments. Abbreviations: metagenome (WGS), metatranscriptome (WTS).

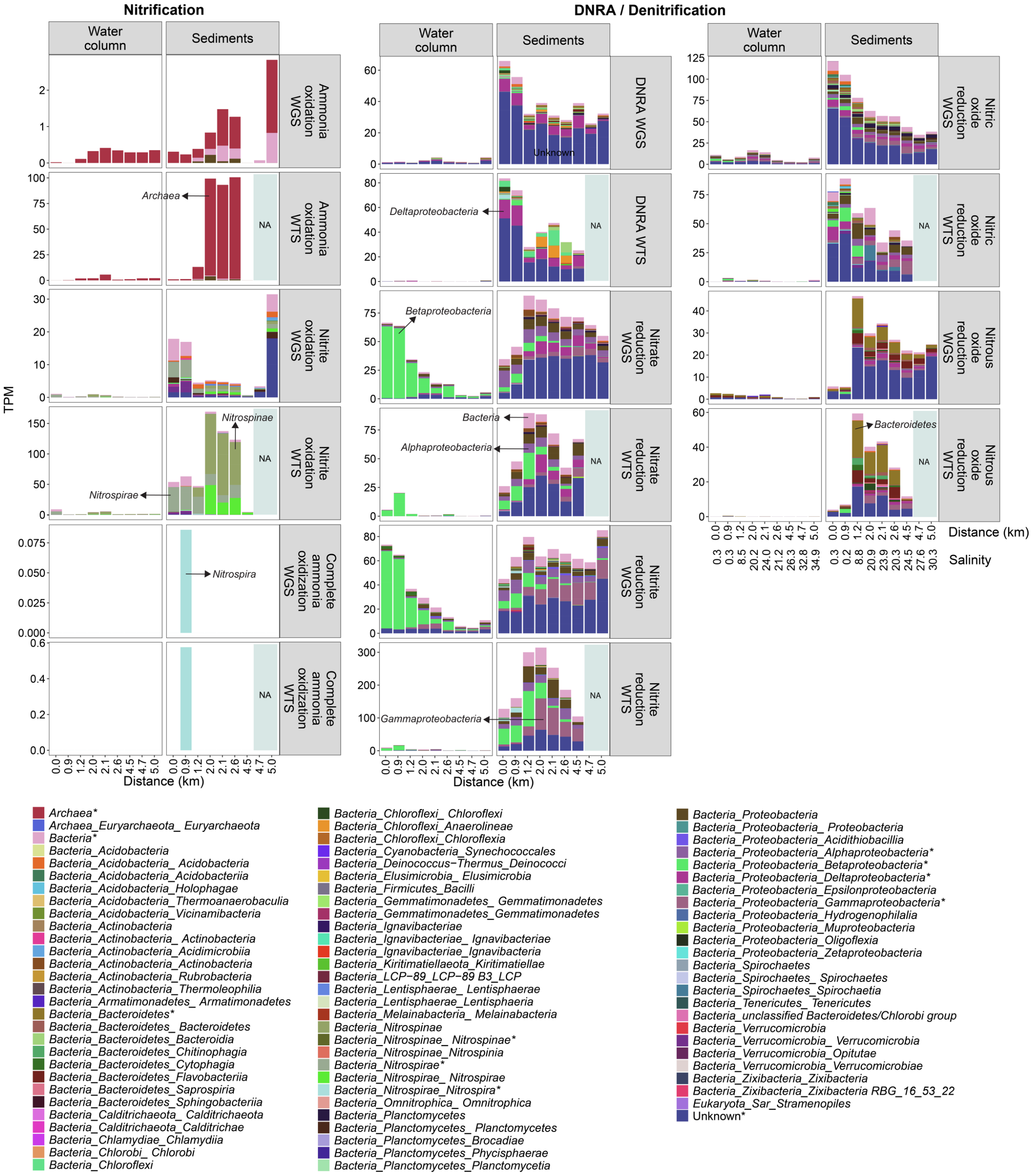

Supplementary Figure 8. Stacked bar plot showing the distribution of taxon-specific genes and transcripts (genes/transcripts per million mapped reads, TPM) involved in **nitrification, denitrification and dissimilatory nitrate reduction to ammonium (DNRA)** across the study sites. NA indicates that no transcriptomic data are available for marine sediments. Abbreviations: metagenome (WGS), metatranscriptome (WTS).

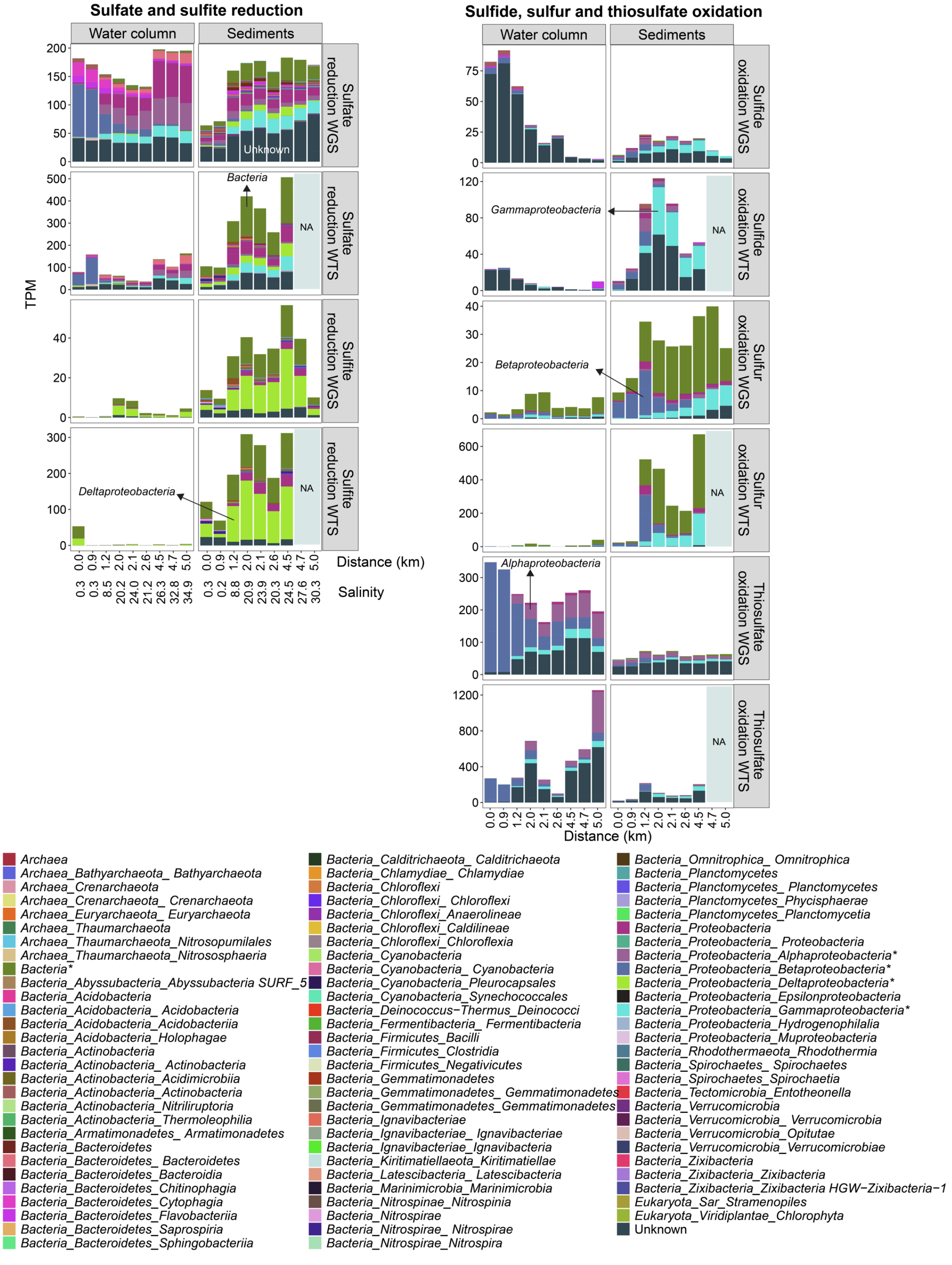

Supplementary Figure 9. Stacked bar plot showing the distribution of taxon-specific genes and transcripts (genes/transcripts per million mapped reads, TPM) involved in **sulfate and sulfite reduction and sulfur, sulfite and thiosulfate oxidation** across the study sites. NA indicates that no transcriptomic data are available for marine sediments. Abbreviations: metagenome (WGS), metatranscriptome (WTS).

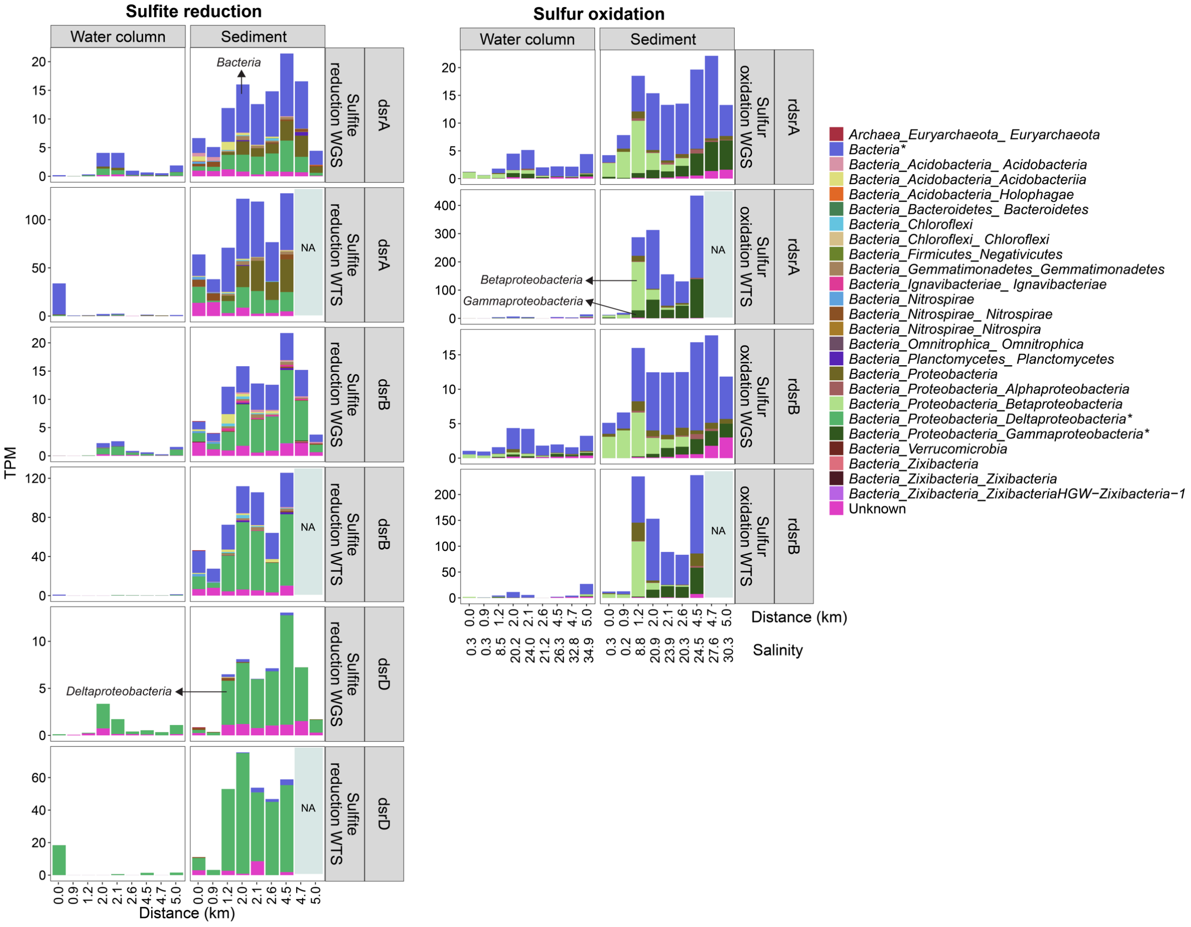

Supplementary Figure 10. Stacked bar plot showing the distribution of taxon-specific genes and transcripts (genes/transcripts per million mapped reads, TPM) involved in **sulfite reduction (dsr) and sulfur oxidation (rdsr)** across the study sites. NA indicates that no transcriptomic data are available for marine sediments. Abbreviations: metagenome (WGS), metatranscriptome (WTS).

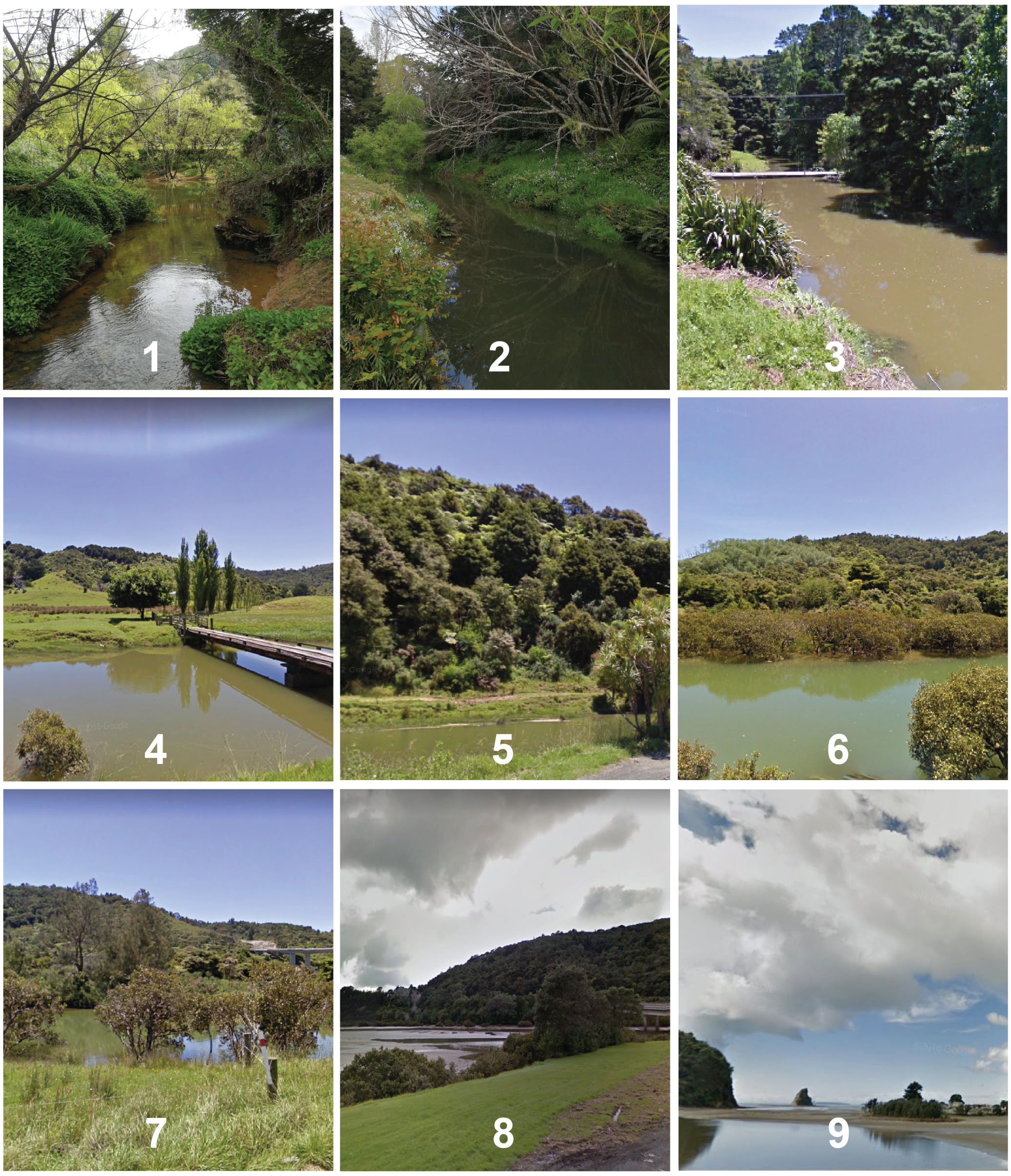

Supplementary Figure 11. Images of sampling sites along the Waiwera River and Estuary. Numbers depict sites progressing from the river (1, 2), estuary (3, 4, 5, 6, 7) to coastal marine (8, 9).

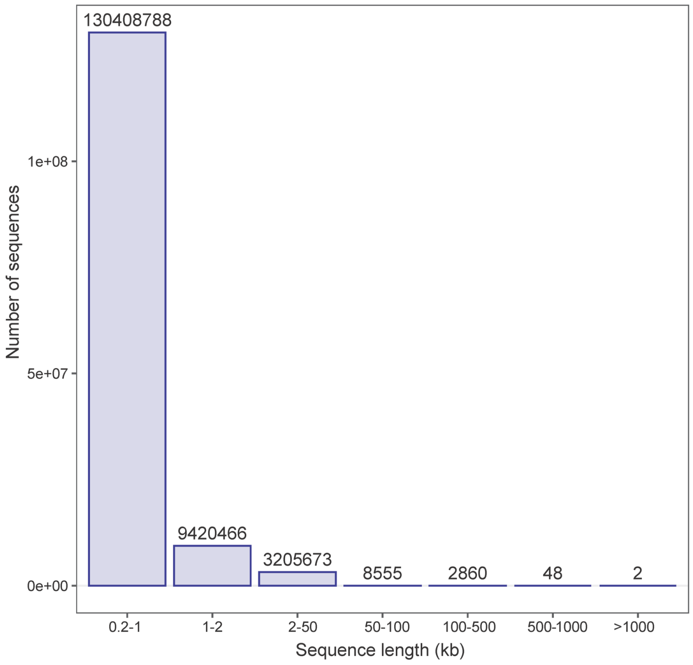

Supplementary Figure 12. Histogram of the size distribution of DNA sequences used for downstream analysis. The average sequence length was 638 bp and ranged from 121 bp to 1,178 kb across samples (n=36). The N50 (shortest contig length needed to cover 50% of the metagenome) was 602 bp, averaged across samples. Overall, 99.8 Mbp (0.1 %) of total sequence length was in the ≤200 bp fraction, 64.1 Gbp (70.2 %) in the <1 kbp fraction, and 27.1 Gbp (29.7 %) in the ≥1 kbp fraction.
